# Atomic Scale Design of MXenes and Their Parent Materials—From Theoretical and Experimental Perspectives

**DOI:** 10.1021/acs.chemrev.3c00241

**Published:** 2023-11-17

**Authors:** Jie Zhou, Martin Dahlqvist, Jonas Björk, Johanna Rosen

**Affiliations:** Materials Design Division, Department of Physics, Chemistry, and Biology (IFM), Linköping University, SE-581 83 Linköping, Sweden

## Abstract

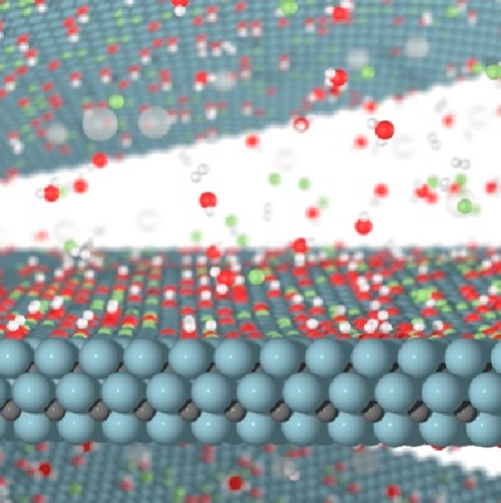

More than a decade after the discovery of MXene, there has been a remarkable increase in research on synthesis, characterization, and applications of this growing family of two-dimensional (2D) carbides and nitrides. Today, these materials include one, two, or more transition metals arranged in chemically ordered or disordered structures of three, five, seven, or nine atomic layers, with a surface chemistry characterized by surface terminations. By combining M, X, and various surface terminations, it appears that a virtually endless number of MXenes is possible. However, for the design and discovery of structures and compositions beyond current MXenes, one needs suitable (stable) precursors, an assessment of viable pathways for 3D to 2D conversion, and utilization or development of corresponding synthesis techniques. Here, we present a critical and forward-looking review of the field of atomic scale design and synthesis of MXenes and their parent materials. We discuss theoretical methods for predicting MXene precursors and for assessing whether they are chemically exfoliable. We also summarize current experimental methods for realizing the predicted materials, listing all verified MXenes to date, and outline research directions that will improve the fundamental understanding of MXene processing, enabling atomic scale design of future 2D materials, for emerging technologies.

## 1 Introduction

**Figure 1:**
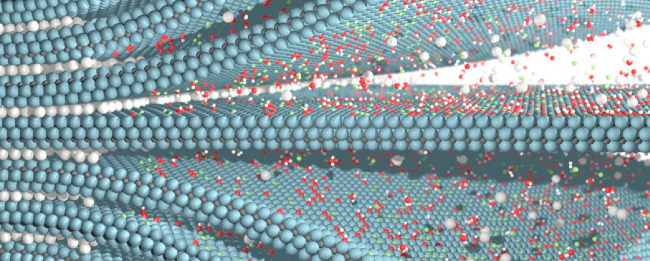
Schematic of the selective etching procedure that converts a 3D material, here a MAX phase (M_3_AX_2_), into a 2D MXene. The etching is given by chemical reactions through which the A elements are removed, and the exposed metal surfaces are terminated by chemical species inherent from the choice of 3D to 2D conversion method. Blue and gray atoms represent the M and X elements, respectively, while the large white atoms in between MXene layers are A elements. Termination elements are exemplified by fluorine, oxygen, and hydroxyl groups, while species in the solution are represented by fluorine, removed A elements, formed hydrogen gas, and water molecules. Fluorine, oxygen, and hydrogen are represented by green, red, and (small) white atoms, respectively. Note that the real etching procedure is more complex than what can be depicted in this schematic.

MXenes are a class of two-dimensional (2D) materials that are composed of transition metal carbides, nitrides, or carbonitrides with the general formula M_*n*+1_X_*n*_T_*x*_, where M is a transition metal, X is carbon or nitrogen, and T_*x*_ represents surface terminations.^1^ The structure of a traditional MXene can be described as *n*+1 M layers, packed into a hexagonal lattice, interleaved with *n* layers of carbon or nitrogen, occupying the octahedral sites between the adjacent M layers. MXenes are generally obtained by selectively etching the A layers (e.g., Al, Si, Ga) from MAX phases,^2,3^ resulting in layered stacks of 2D material. A schematic overview of the etching procedure is depicted in Figure 1.

In 2011, the first MXene was discovered when Naguib et al. realized Ti_3_C_2_T_*x*_ by selective etching of monatomic Al layers from the Ti_3_AlC_2_ MAX phase precursor in hydrofluoric (HF) acid.^1,4^ The material had a mixture of surface terminations—O, OH, and F—inherent from the etching process, and the unique combination of hydrophilicity and a high metallic conductivity, together with an ability to intercalate ions, motivated the exploration of MXene for energy storage applications.^5^ Since then, a multitude of MXenes have been discovered, with tunable properties originating from both a rich chemistry of the backbone material as well as interchangeable surface terminations, ranging from intermixed species to single element terminations (e.g., Br, I).^6,7^

Today the number of unique MXene compositions is over 50. Synthesized MXenes span over 11 different transition metals, from Sc and Y in group 3 to Cr, Mo, and W in group 6, and can be composed of one, two, or more metals (up to high-entropy material), as well as one or two elements on the X site. Moreover, double M MXenes can have the metals randomly distributed in a chemically disordered solid solution, typically denoted (M′, M′′)_*n*+1_X_*n*_T_*x*_, or be chemically ordered through out-of-plane ordering of individual layers of M′ and M′′ in a sandwich structure (*o*-MXene), referred to as M′_2_M′′C_2_T_*x*_ or M′_2_M′′_2_C_3_T_*x*_. Since 2017, there are also MXenes characterized by in-plane chemical order, *i*-MXenes, for which the general formula to date is M′_4/3_M′′_2/3_CT_*x*_, where the order is given through either in-plane ordering of the two M elements or ordered vacancies after selective removal of the A element as well as M′′ upon chemical exfoliation of the 3D precursor.

While the first MXene was initially tested for supercapacitor applications, and as the MXene family started growing, there was a surge of research activities that led to a fast expansion of the field. Today, MXenes are being explored for a variety of applications, ranging from structural composites to optoelectronics (electromagnetic interference shielding, wireless communication, electrochromics), electrocatalysis (separation of gases, water purification, chemical sensing), and medicine (dialysis, photothermal therapy). Programmable morphology structures, lasers and photonics, environmental remediation, memory devices and bioinformatics are among other emerging applications of MXenes, as summarized in a recent review.^8^

Property tailoring for a specific application requires precise control of the materials composition and structure. In this regard, a combined theoretical–experimental approach is a powerful tool for materials development. While the research field now sees an increase in high-throughput simulations and emerging machine learning methods for accelerated materials discoveries, different approaches have been used for predictive explanatory simulations in this area, with diverging results. Rigorous methods need to be applied in order to not flood the community with theoretical predictions that cannot be experimentally realized. Furthermore, method development is required, and for the topic of the present Review, this is with respect both for procedures for predicting if a material can be exfoliated or not and for experimental methods allowing the realization of the predicted materials (ideally scalable methods allowing sustainable processing).

In this Review, we critically assess the field of atomic scale design and synthesis of 2D MXenes and their 3D precursors. Since traditional MXene synthesis requires a stable 3D compound to be selectively etched, further expansion of the MAX phase family (or related laminated materials) is required to correspondingly expand the family of MXenes. We discuss theoretical methods for predicting MXene precursors, from comparing energies such as formation energy (with respect to constituent atoms in their ground-state crystal structure) and formation enthalpy (with respect to its competing phases) to machine learning attempts. As will be shown, formation enthalpy is a good indicator for predicting the stability of already synthesized MAX phases and can be used to guide synthesis attempts of new ones. In addition, the use of formation enthalpy for stability evaluations also gives valuable knowledge that could be used for designing synthesis attempts to avoid the formation of unwanted secondary phases. It will be shown that stability predictions beyond ternary MAX phases, such as mixing multiple metals, require multiple aspects to be considered, such as how the mixing elements are distributed (order or disorder) and the impact from temperature.

We also scrutinize different methods for predicting MXene synthesizability, with particular focus on methodologies for evaluating the possibility of converting a MAX phase into a 2D MXene by selective etching. As will be seen, single layer MXenes, as other freestanding layers of 2D materials, are not thermodynamically stable, and thermodynamic stability cannot be used as an indicator of synthesizability. Instead, it is necessary to evaluate the processes leading from a 3D to a 2D material, by analysis of the exfoliation energy (the reaction energy of going from 3D to 2D). The peculiar case of MXene synthesis, compared to the synthesis of most other 2D materials from 3D precursors, is the change in chemical composition from reactants to products (due to the removal of the A layer and the anchoring of termination groups to the resulting MXene surface), which requires the exchange of atoms with a reservoir, necessitating chemical potentials entering the exfoliation energy. As will be shown, the common description of mechanical exfoliation (in which the MXene and A elements are separated from each other without involving reactions with other substances) always results in the exfoliation being thermodynamically unfavorable and cannot be used to describe MXene synthesis. Instead, it is necessary to make a more accurate assessment of the chemical environment in which the etching takes place, and to compare competing processes during the initial stages of the etching. It should be stressed that this part of the review focuses on theoretical studies attempting at understanding or predicting synthesis protocols of MXenes. Consideration of theoretical studies solely reporting properties of MXenes is beyond the scope of the review.

Once a hypothetical material is predicted, it is crucial to have suitable methods for experimental verification. We therefore also summarize current experimental methods for MXenes synthesis, including traditional top-down approaches as well as emerging techniques allowing direct bottom-up synthesis or chemical vapor deposition (CVD) growth without a 3D precursor. The differences in morphology and terminations of MXenes produced by current experimental methods are also summarized. The Review provides a comprehensive list of all MXenes synthesized to date, though after scrutinizing the literature, we only include those that are experimentally verified and those that can be considered as 2D materials, in single or multilayer form. While surface terminations are important for the materials properties, we only consider surface terminations inherent to the synthesis procedures, excluding attainable modifications achieved through postsynthesis procedures. Finally, we outline research directions that will improve the fundamental understanding of MXenes processing, to enable atomic scale design and discovery of future 2D materials, for expanding the family and adding new compositions and structures.

## 2 MAX Phases—Main Precursor for MXenes

MXenes are typically synthesized through a top-down approach, starting from a layered three-dimensional crystalline material known as MAX phases, where the A layer is selectively removed while layers of transition metal carbides or nitrides (M_*n*+1_X_*n*_) are intact. MAX phases were discovered in 1960 by Rohde and Kudielka with the synthesis of Ti_2_SC and Zr_2_SC.^9^ A few years later, 34 additional MAX phases were reported by Jeitschko, Nowotny, and Benesovksy,^10−17^ and the family of MAX phases was established. In the 1990s, a renewed interest in these materials was spurred by the pioneering work of Barsoum and El-Raghy who synthesized relatively phase-pure samples of Ti_3_SiC_2_ and demonstrated a material with a unique combination of metallic and ceramic characteristics.^18−22^ MAX phases are generally synthesized by reactive sintering of elemental powders but can also be grown in, e.g., thin film form.^3,23^ Today, there are more than 300 reported MAX phases with unique elemental combinations composed of 116 ternary and over 200 multinary MAX phase compounds, the latter by mixing multiple elements on the M (by far most common), A, and/or X sites.^24^

Since MXenes are typically synthesized using a top-down approach, the composition of M_*n*+1_X_*n*_ is inherited from the composition of their precursor MAX phase. Whether there is one or more transition metals and if alloying elements form chemical order or disorder in the 3D laminate is typically translated into the 2D counterpart. Control of the inherent MXene chemistry is therefore given by corresponding control of the chemical composition and arrangement of transition metals and/or X elements in its parent/precursor material, namely, the MAX phases. The following section gives a summary of MAX phases and non-MAX phase compounds synthesized to date that have been successfully transformed into MXenes. We thereafter review pathways for theoretical prediction of novel 3D MXene precursors, which is crucial for the design and discovery of novel MXenes.

### 2.1 Structure and Composition of MXene Parent Materials

**Figure 2:**
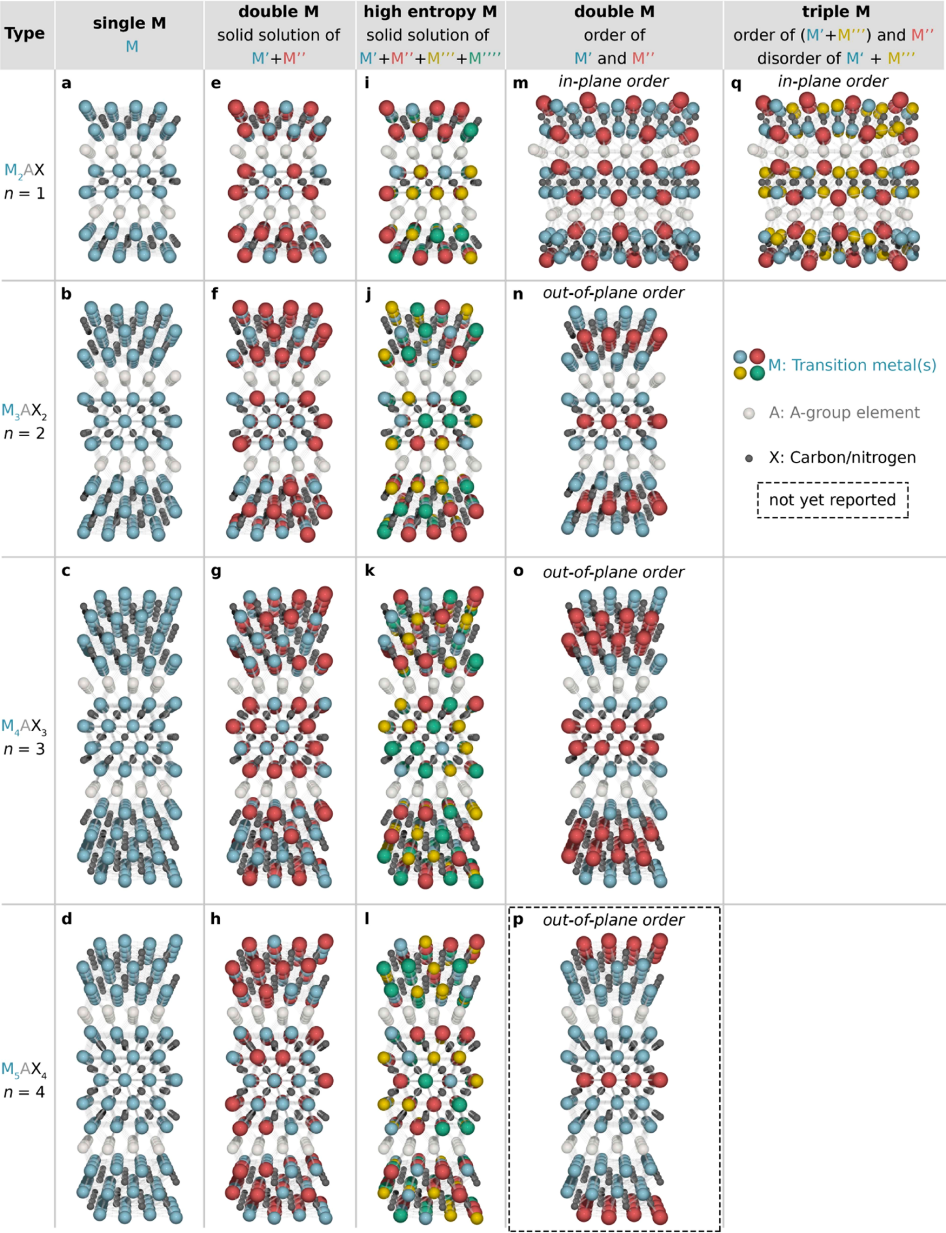
Schematic overview of MAX phase structures used for MXene synthesis. MAX phases have a general formula of M_*n*+1_AX_*n*_ where M is an early transition metal, A is an A-group element, and X is carbon and/or nitrogen. The value of *n* typically ranges from 1 to 4, depending on the number of transition metal carbide and/or nitride layers M_*n*+1_X_*n*_ present in the structure of the MAX phase. The metal (M) sites of MAX phases can be occupied with (a–d) one, (e–h, m–p) two, or (i–l, q) multiple transition metal atoms (e–l) being randomly arranged in a solid solution or (m–q) forming a chemically ordered atomic configuration with the metals occupying specific lattice sites. Dashed rectangles indicate structures not yet reported.

MAX phases are hexagonal layered materials of a *P*6_3_/*mmc* space group symmetry which adheres to the general M_*n*+1_AX_*n*_ stoichiometry, where *n* = 1, 2, 3, etc. determines the thickness of the transition metal carbide and/or nitride layers (M_*n*+1_X_*n*_) present in the structure of the MAX phase; see Figure 2a–d. Its layered structure can further be described as layers of M_6_X octahedra separated by a layer of A, where A is located in the center of a trigonal prism of M. There is a short notation which directly reflects the composition of M, A, and X: 211 (*n* = 1) for M_2_AX, 312 (*n* = 2) for M_3_AX_2_, and 413 (*n* = 3) for M_4_AX_3_.

#### 2.1.1 Single Metal Precursors

The traditional MAX phase is a ternary compound composed of one transition metal M; an element denoted A, since it originally referred to A-group elements such as Al, Ga, In, Si, Ge, Sn, S, and P, while nowadays the A site can be occupied by Fe, Co, Ni, Cu, Zn, Ir, Au, Se, and Te; and with carbon, nitrogen, and most recently boron on the X site. In addition, the X site can be partially occupied by O.^25,26^ There are in total 124 ternary MAX phases synthesized to date, and the most common ones have Al as the A layer.^24^ The size of the MAX phase is determined by *n*, and the three most common ones are M_2_AX (*n* = 1, 85 phases), M_3_AX_2_ (*n* = 2, 19 phases), and M_4_AX_3_ (*n* = 3, 8 phases). Schematic crystal structures illustrating these phases are shown in Figure 2a–c. There are also examples of MAX phases where *n* > 4, like Ta_6_AlC_5_ (*n* = 5)^27^ and Ti_7_SnC_6_ (*n* = 6),^28^ though none of these have been converted into MXenes. In addition, there are intergrown MAX phases, which are a combination of M_3_AX_2_ and M_2_AX or M_4_AX_3_, respectively, giving rise to alternating layers of M_*n*+1_X_*n*_ with different *n*, like Ti_5_Al_2_C_3_ and Ti_7_Si_2_C_5_,^29,30^ but these are rare.

Most MXenes are made by selective etching of Al from a MAX phase. Out of the 18 known Al-based ternary MAX phases, only half of them have been successfully converted into MXenes; see a list of all 3D precursors used for MXene synthesis in Table 1. The list demonstrates the need for improved etching procedures for MXene synthesis from MAX phases with A ≠ Al or from materials beyond MAX phases. Examples of such pathways have been shown for Ti_3_SiC_2_^31^ and Ti_3_ZnC_2_^32^ for making Ti_3_C_2_T_*x*_.

**Table 1:** List of Synthesized Precursor Materials Used for Making MXenes

General MXene notation	*n*	Precursor materials	MXene
M_*n*+1_X_*n*_	1	Ti_2_AlC,^17^ Ti_2_ZnC^32^	Ti_2_C^4,32^
		V_2_AlC,^43^ V_2_ZnC^32^	V_2_C^32,44^
		Nb_2_AlC^17^	Nb_2_C^44^
		Mo_2_Ga_2_C^33^	Mo_2_C^34,35^
		Ta_2_AlC^17^	Ta_2_C^45^^,^^a^
		Ti_2_AlN^13^	Ti_2_N^46^
		Ammoniating V_2_CT_*x*_ MXene	V_2_N^47^
	2	Ti_3_AlC_2_,^48^ Ti_3_SiC_2_,^49^ Ti_3_ZnC_2_^32^	Ti_3_C_2_^1,31,32,50^
		Zr_3_Al_3_C_5_^36^	Zr_3_C_2_^39^
		Hf_3_[Al(Si)]_4_C_6_^37,38^	Hf_3_C_2_^38^
	3	Ta_4_AlC_3_^51^	Ta_4_C_3_^4^
		Nb_4_AlC_3_^52^	Nb_4_C_3_^55^
		V_4_AlC_3_^53^	V_4_C_3_^56^
		Ti_4_AlN_3_^54^	Ti_4_N_3_^57^
(M′,M′′)_*n*+1_X_*n*_, disordered solid solution on M site	1	(Ti_1–*x*_Nb_*x*_)_2_AlC^58^	(Ti_1–*x*_Nb_*x*_)_2_C^4,60^
		(Ti_1–*x*_V_*x*_)_2_AlC^58^	(Ti_1–*x*_V_*x*_)_2_C^60^
		(V_1–*x*_Nb_*x*_)_2_AlC^59^	(V_1–*x*_Nb_*x*_)_2_C^60^
		(V_1–*x*_Cr_*x*_)_2_AlC^58^	(V_1–*x*_Cr_*x*_)_2_C^4^
	2	(V_1–*x*_Cr_*x*_)_3_AlC_2_^61^	(V_1–*x*_Cr_*x*_)_3_C_2_^4^
		(Ti_1–*x*_V_*x*_)_3_AlC_2_^59^	(Ti_1–*x*_V_*x*_)_3_C_2_^63^
		(Ta_1–*x*_Ti_*x*_)_3_AlC_2_^62^	(Ta_1–*x*_Ti_*x*_)_3_C_2_^62^
	3	(Nb_0.8_Ti_0.2_)_4_AlC_3_^64^	(Nb_0.8_Ti_0.2_)_4_C_3_^64^
		(Nb_0.8_Zr_0.2_)_4_AlC_3_^65^	(Nb_0.8_Zr_0.2_)_4_C_3_^64^
		(Mo_1–*x*_V_*x*_)_4_AlC_3_^66^	(Mo_1–*x*_V_*x*_)_4_C_3_^66^
		(Nb_1–*x*_Ta_*x*_)_4_AlC_3_ (*x* ≥ 0.75)^67^	(Nb_0.875_Ta_0.125_)_4_C_3_^68^
		(Nb_0.975_W_0.025_)_4_AlC_3_^68^	(Nb_0.975_W_0.025_)_4_C_3_^68^
		(Ti_*x*_Ta_1–*x*_)_4_AlC_3_^69^	(Ti_*x*_Ta_1–*x*_)_4_C_3_^69^
	4	(Mo_1–*x*_V_*x*_)_5_AlC_4_^70^	(Mo_1–*x*_V_*x*_)_5_C_4_^70^
(M′,M′′,M′′′,M′′′′)_*n*+1_X_*n*_, disordered solid solution high-entropy MXene	1	(Ti_1/5_V_1/5_Zr_1/5_Nb_1/5_Ta_1/5_)_2_AlC^71^	(Ti_0.30_V_0.20_Zr_0.10_Nb_0.17_Ta_0.23_)_2_C^71^
		(Ti_1/3_V_1/6_Zr_1/6_Nb_1/6_Ta_1/6_)_2_Al(C_1–*x*_N_*x*_)^72^	(Ti_0.28_V_0.20_Zr_0.09_Nb_0.20_Ta_0.24_)_2_(C_1–*x*_N_*x*_)^72^
		(Ti,V,Nb,Ta)_2_AlC,^73^ (Ti_0.25_V_0.25_Nb_0.25_Ta_0.25_)_2_GaC^74^	(Ti,V,Nb,Ta)_2_C^73^
		(Ti,V,Nb,Hf,Ta)_2_AlC^73^	(Ti,V,Nb,Hf,Ta)_2_C^73^
		(Ti_0.33_V_0.33_Nb_0.33_)_2_GaC^74^	(Ti_0.33_V_0.33_Nb_0.33_)_2_C,^74^ (Ti_0.2_V_0.2_Nb_0.2_Mo_0.2_Ta_0.2_)_2_C^74^
		(Ti_0.2_V_0.2_Nb_0.2_Mo_0.2_Ta_0.2_)_2_GaC^74^	
	2	(Ti_0.67_V_0.30_Cr_0.03_)_3_AlC_2_^75^	(Ti_0.67_V_0.30_Cr_0.03_)_3_C_2_^75^
	3	(Ti_0.25_V_0.25_Nb_0.25_Mo_0.25_)_4_AlC_3_^76^	(Ti_0.225_V_0.25_Nb_0.25_Mo_0.225_)_4_C_3_^76^
		(Ti_0.25_V_0.25_Cr_0.25_Mo_0.25_)_4_AlC_3_^76^	(Ti_0.275_V_0.275_Cr_0.225_Mo_0.25_)_4_C_3_^76^
		(Ti_1.27_V_0.19_Cr_0.013_Nb_0.27_Ta_0.27_)_4_AlC_3_^77^	(Ti_0.275_V_0.175_Cr_*x*_Nb_0.25_Ta_0.15_)C_3_^77^
	4	(Ti_0.22_V_0.24_Cr_0.16_Nb_0.20_Mo_0.18_)_5_AlC_4_^78^	(Ti_0.22_V_0.24_Cr_0.16_Nb_0.20_Mo_0.18_)_5_C_4_^78^
M_*n*+1_(X′,X′′)_*n*_, disordered solid solution on X site	1	Ti_2_Al(C_1–*x*_N_*x*_)^79,80^	Ti_2_(C_0.5_N_0.5_)^6^
		Ammoniating Mo_2_CT_*x*_ MXene	Mo_2_(C_1–*x*_N_*x*_)^47^
	2	Ti_3_Al(C_1–*x*_N_*x*_)_2_^81^	Ti_3_(C_1–*x*_N_*x*_)_2_^4^
		Ti_3_Al(C_0.7_O_3_)_2_^26^	Ti_3_(C_0.7_O_3_)_2_^26^
M_1.33_X, disordered vacancies	1	(Nb_0.67_Sc_0.33_)_2_AlC^82^	Nb_1.33_C^82^
		(V_1–*x*_Sc_*x*_)_2_AlC (*x* ≤ 0.05)^83^	V_2–*x*_C^83^
M_4/3_X, ordered vacancies	1	Mo_4/3_Sc_2/3_AlC,^84^ Mo_4/3_Y_2/3_AlC^85^	Mo_4/3_C^84,90,91^
		Mo_4/3_RE_2/3_AlC (RE = Gd, Tb, Dy, Ho, Er, and Y)^86^	
		W_4/3_Sc_2/3_AlC,^87,88^ W_4/3_Y_2/3_AlC,^87,88^	W_4/3_C^87−89^
		W_4/3_RE_2/3_AlC (RE = Gd, Tb, Dy, Ho, Er, and Tm)^89^	
M′_4/3_M′′_2/3_X, in-plane chemical order	1	Mo_4/3_Y_2/3_AlC^85^	Mo_4/3_Y_2/3_C^90^
M′_2/3_M′′_2/3_X, ordered vacancies and disordered solid solution of M′ and M′′	1	W_2/3_Mo_2/3_RE_2/3_AlC (RE = Y, Gd, Tb, Dy, Ho, and Er)^92^	W_2/3_Mo_2/3_C^92^
M′_2_M′′_*n*–1_X_*n*_, out-of-plane chemical order	2	Mo_2_ScAlC_2_^93^	Mo_2_ScC_2_^93^
		Mo_2_TiAlC_2_^94^	Mo_2_TiC_2_^96^
		Cr_2_TiAlC_2_^95^	Cr_2_TiC_2_^96^
	3	Mo_2_Ti_2_AlC_3_^97^	Mo_2_Ti_2_C_3_^96^
		Mo_2_Nb_2_AlC3^98^	Mo_2_Nb_2_C_3_^98^

a Claimed to be synthesized, but little or no experimental evidence.

**Figure 3:**
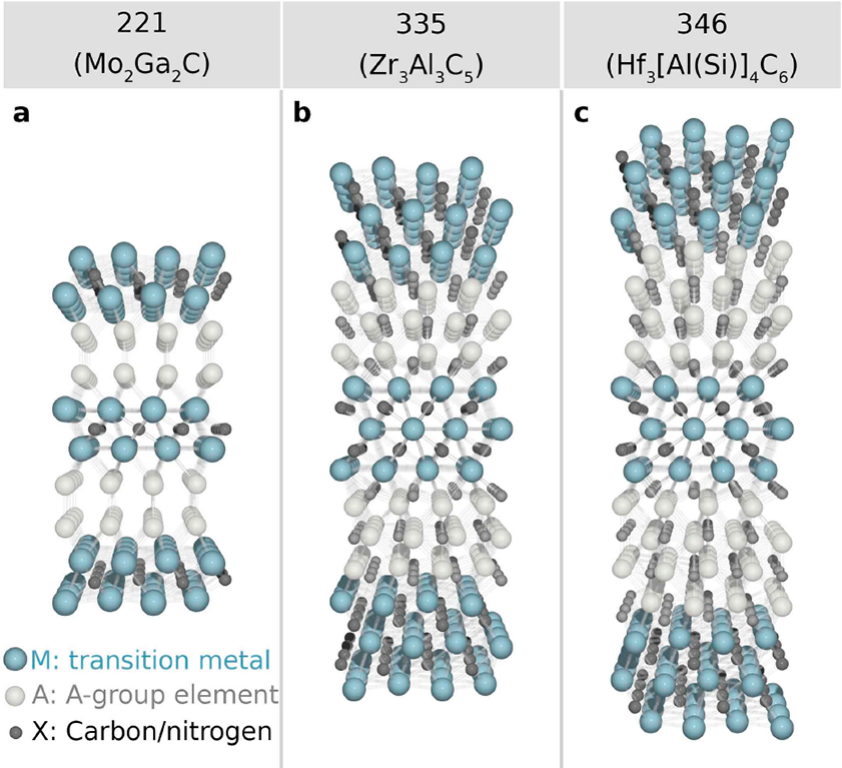
MAX phase-like compounds used as precursors for MXenes; crystal structures of (a) Mo_2_Ga_2_C, (b) Zr_3_Al_3_C_5_, and (c) Hf_3_[Al(Si)]_4_C_6_, respectively.

A material closely resembling a MAX phase is Mo_2_Ga_2_C,^33^ illustrated in Figure 3a and composed of a double layer of Ga interleaving Mo_2_C. The Ga layers have been selectively etched to produce Mo_2_C MXene,^34,35^ as attempts to convert the closely related Mo_2_GaC MAX phase to a 2D material have failed. Other examples of the synthesis of MXenes from non-MAX precursors are Zr_3_Al_3_C_5_^36^ and Hf_3_[Al(Si)]_4_C_6_,^37,38^ illustrated in Figure 3b–c. Unlike MAX phases, which have M_*n*+1_X_*n*_ units separated by a single A layer, these materials are composed of units of M_3_C_2_ interleaved by aluminum carbide (Al_3_C_3_ or [Al(Si)]_4_C_6_) layers, which can be selectively etched to synthesize Zr_3_C_2_T_*x*_ and Hf_3_C_2_T_*x*_ MXenes.^38,39^ These materials belong to a family of layered transition metal carbides, much like MAX phases, with a general composition of M_*n*_Al_3_C_*n*+2_ and M_*n*_Al_4_C_*n*+3_, where M is typically Zr or Hf and *n* = 1, 2, or 3.^40−42^ In total, 23 single metal MXenes have been reported to date (Table 1).

Going beyond ternary MAX phases by considering the possibility for mixing elements on any of the M, A, and X sites gives rise to more than 200 unique MAX phase elemental combinations.^24^ MAX phases with more than one metal exist in two forms: (i) as a solid solution of two or more metals distributed randomly within and across the metal layers, and (ii) as a chemically ordered compound where two metals are distributed in a chemically ordered configuration.

#### 2.1.2 Double-Metal Precursors (Solid Solutions)

Double-metal MAX phases have two metals, M′ and M′′, which in a random arrangement form a solid solution within the M layers. These are schematically illustrated in Figure 2e–h. The first report of such phases was published in 1980, when Schuster and co-workers discovered (Ti_1–*x*_V_*x*_)_2_AlC and (V_1–*x*_Cr_*x*_)_2_AlC.^58^ Both phases were later transformed into (Ti_1–*x*_V_*x*_)_2_CT_*x*_ and (V_1–*x*_Cr_*x*_)_2_CT_*x*_ MXenes.^4,60^ All MAX phases with double-metal solid solution that have been converted into MXene are listed in Table 1 and encompass *n* from 1 (e.g., (V_1–*x*_Nb_*x*_)_2_AlC)^60^ to 4 ((Mo_1–*x*_V_*x*_)_5_AlC_4_).^70^ Note that (Mo_1–*x*_V_*x*_)_5_AlC_4_ does not adhere to the traditional MAX phase structure as it shows twinning in the M layers,^70^ as illustrated in Figure 2h. To date, a total of 55 double-metal solid solution MAX phases have been discovered, distributed over 30 (M′_1–*x*_M′′_*x*_)_2_AlC, 11 (M′_1–*x*_M′′_*x*_)_3_AlC_2_, 11 (M′_1–*x*_M′′_*x*_)_4_AlC_3_, and 3 (M′_1–*x*_M′′_*x*_)_5_AlC_4_. Fifteen of them have been converted to MXene. Notably, almost 50% of the 14 double-metal solid solution MXenes known are (M′_1–*x*_M′′_*x*_)_4_C_3_T_*x*_.

#### 2.1.3 High-Entropy Metal Precursors (Solid Solutions)

In recent years there has been progress in the synthesis of MAX phases with more than two metals.^71,72,74−78,99−107^ These so-called high-entropy MAX phases, schematically illustrated in Figure 2i–l, are composed of three to five metals (M′ + M′′ + M′′′ + ...) forming multielement solid solutions across the M layers. Corresponding MXenes have been demonstrated for *n* = 1,^71−74^*n* = 2,^75^*n* = 3,^76,77^ and *n* = 4.^78^ The multielemental high-entropy MAX and MXenes will enable pathways for tuning the phase composition and ultimately the materials properties.

#### 2.1.4 Double-Metal Precursors (Chemical Order)

Another example which has initiated a significant expansion of the MAX phase family is double-metal MAX phases where M′ and M′′ occupy specific lattice sites, resulting in chemical order. The first example is when M′ and M′′ atoms occupy separate atomic planes, with M′ next to the A layers and M′′ in the inner part of the M_*n*+1_X_*n*_ layers, giving rise to out-of-plane ordering. The short notation used for this type is *o*-MAX, and it is only attainable for *n* ≥ 2 (Figure 2n–p). The first phase of this type was reported in the form of Cr_2_TiAlC_2_,^95^ soon followed by other out-of-plane ordered MAX phases such as Cr_2_VAlC_2_, Mo_2_TiAlC_2_, Mo_2_ScAlC_2_, Mo_2_Ti_2_AlC_3_, and Mo_2_Nb_2_AlC_3_.^93,94,97,98,108^ The out-of-plane order of M′ and M′′ is retained upon etching, and the corresponding *o*-MXene composition is M′_2_M′′X_2_T_*x*_ and M′_2_M′′_2_X_3_T_*x*_.

The second example is when M′ and M′′ order within a metal layer, typically referred to as *i*-MAX. Its structure is illustrated in Figure 2m, where M′′ have a larger atomic size and therefore extended toward the A layer, as first demonstrated for Mo_4/3_Sc_2/3_AlC.^84^ Since then, 52 additional *i*-MAX phases have been reported^24^ where M′′ is Sc, Y, Zr, or a rare earth (RE) element and M′ is Mo, W, V, or Cr.^83,85,86,88,89,92,109−113^ Corresponding 2D derivatives are denoted *i*-MXenes and have a general formula M′_4/3_M′′_2/3_XT_*x*_, where M′′ can be etched or retained upon chemical exfoliation, resulting in ordered vacancies or a chemically ordered metal configuration, respectively.

#### 2.1.5 Double X Precursors (Solid Solutions)

In addition to carbide and nitride MAX phases, there are carbonitride and oxycarbide MAX phases, with carbon and nitrogen or carbon and oxygen being randomly arranged on the X site. These are rare and have only been demonstrated for Ti_2_Al(C_1–*x*_N_*x*_),^79,80^ V_2_Ga(C_1–*x*_N_*x*_),^114^ Zr_2_Se(B_1–*x*_Se_*x*_) (0 < *x* < 0.97),^115^ Ti_3_Al(C_0.5_N_0.5_)_2_,^81,116^ Ti_4_Al(C_1−*x*_N_*x*_)_3_, (Ti_0.333_V_0.167_Zr_0.167_Nb_0.167_Ta_0.167_)_2_Al(C_*x*_N_1–*x*_),^72^ Ti_2_Al(C_0.5_O_0.5_),^25^ and Ti_3_Al(C_0.7_O_0.3_)_2_.^26^ Corresponding MXene carbonitrides includes Ti_3_(C_1–*x*_N_*x*_)_2_T_*x*_^4^ Ti_4_(C_1−*x*_N_*x*_)_3_, and Ti_2_(C_0.5_N_0.5_)T_*x*._^6^ Another example is Mo_2_(C_1–*x*_N_*x*_)T_*x*_,^47^ which was obtained through ammoniating Mo_2_C MXene, resulting in partial substitution of carbon for nitrogen. There is one example of an oxycarbide MXene, Ti_3_(C_0.7_O_0.3_)_2_.^26^

### 2.2 Finding Novel MAX Phase Precursor Materials

The discovery of new MAX phase structures and compositions, and precise control of the distribution of alloying elements, are critical components for continued MXenes development and for expanding attainable properties for use in various applications. Here computational predictions can serve as a valuable tool for identifying precursor candidates likely to be synthesized. We will here summarize different approaches used in the literature for evaluating the stability of MAX phases, starting with coarse-grained ones and then adding more complexity.

#### 2.2.1 Formation Energy—Providing Limited Guidance for Materials Synthesis

Predicting the stability of a MAX phase includes a comparison of energies. In its simplest form, this is done by comparing the calculated MAX phase energy with its constituent atoms in their ground-state crystal structure. This is known as the formation energy, Δ*E*_f_, and for a ternary M_*n*+1_AX_*n*_ phase is given by
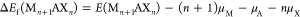
1where *E*(M_*n*+1_AX_*n*_) is the calculated total energy of the M_*n*+1_AX_*n*_ phase, μ_*i*_ is the chemical potential of element *i*, and *n* is typically 1, 2, or 3. The standard convention is to consider the chemical potential of each species as the total energy of the elemental ground-state crystal structure. With this choice, Δ*E*_f_ is valid only for 0 K.

**Figure 4:**
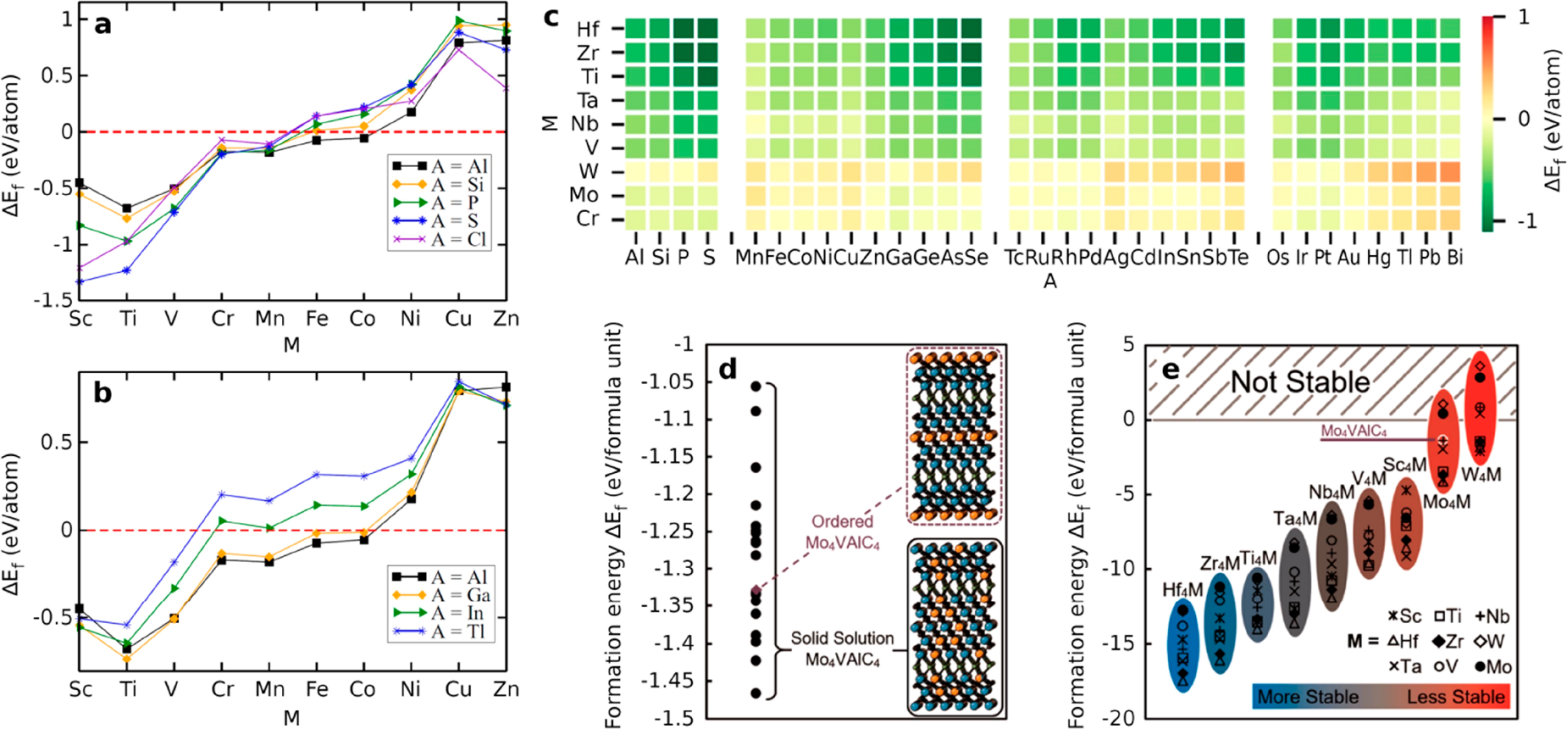
(a–b) Formation energies Δ*E*_f_ for 90 M_2_AC MAX phases as a function of M when A changes across (a) group and (b) period in the periodic table of elements. (c) Δ*E*_f_ for 288 M_2_AC phases displayed as a heatmap for 8 metals and 32 different elements at the A site. (d) Δ*E*_f_ for solid solution Mo_4_VAlC_4_, or (Mo_0.8_V_0.2_)_5_AlC_4_, configurations (black dots) compared to ordered Mo_4_VAlC_4_ (purple diamond) with V occupying the central M layer. (e) Δ*E*_f_ for 72 ordered M′_4_M″AlC_4_ MAX phases. Most are found with Δ*E*_f_ < 0 except for some Mo and W compositions. (a–e) Reproduced with permission from refs (70, 117, and 127). Copyright 2020 American Chemical Society, 2023 Elsevier, and 2019 American Physical Society, respectively.

However, it is important to note that Δ*E*_f_ is not a particularly appropriate indicator for stability since it only accounts for decomposition of a MAX phase into its elemental phases, leading to a severe overestimation of the stability. Figure 4 shows a few examples where Δ*E*_f_ have been used to evaluate the stability of MAX phases. Figure 4c shows Δ*E*_f_ for 288 M_2_AC MAX phases where a majority are found with Δ*E*_f_ < 0.^117^ Note that Δ*E*_f_ may indeed show interesting trends within and across groups for M and A; however, from a phase stability point of view, it bears little information to be used for predicting synthesizable compounds. While the formation energy is negative for 222 compounds, only 50 of the 288 considered MAX phases have been synthesized.^24^ In Figure 4a–b we again find clear trends in Δ*E*_f_ for an increase in the valence of M, where M = Sc, Ti, and V are among the M_2_AC phases that are most stable with respect to their elemental constituents. However, the trend shows little resemblance with calculated thermodynamic stability when compared to compounds beyond elemental phases, i.e., competing phases such as binaries and ternaries; see section 2.2.2 for more details. Figure 4e shows the formation energy accounting for out-of-plane order on the M sites in quaternary MAX phases for different compositions. At first sight, the trend with increasing formation energy when going from Hf_4_MAlC_4_ to W_4_MAlC_4_ may be mistaken as a sign of decreasing stability, or going from more stable to less stable. However, this does not necessarily have to be the case. Taking Ti_4_MAlC_4_ as an example, which in Figure 4e is compared to bulk Ti, M, Al, and C, where there are many stable Ti-based MAX phases that are not considered in such a comparison, and therefore the stability will be severely overestimated when using only the formation energy.^70^ Another example is given in Figure 4d. While Mo_4_VAlC_4_, or rather (Mo_0.8_V_0.2_)_5_AlC_4_ to account for the M intermixing, and its MXene derivative have been synthesized, the presented formation energy in Figure 4d cannot be used to directly conclude whether the investigated phases are stable or not with respect to compounds beyond elemental ones. However, the representation can still serve a purpose when comparing order with disorder. Further examples from the past where formation energy Δ*E*_f_ < 0 has been used as a condition for claiming stability of MAX phases can be found in, e.g., refs (70, 96, and 117−126).

#### 2.2.2 Formation Enthalpy

A more comprehensive approach for assessing the thermodynamic stability of a M_*n*+1_AX_*n*_ phase is to compare its calculated energy with respect to the calculated energy of all possible linear combinations of phases present in the M-A-X material system by solving a linear optimization problem.^128−130^ There are two alternative methodologies for how to select which phases are being considered as competing phases. The first approach is the convex hull construction which takes into account all competing phases, including the M_*n*+1_AX_*n*_ phase of interest. A stable MAX phase, according to this definition, will be part of the convex hull. That is, its energy is compared with itself, and therefore Δ*H*_CHULL_ = 0. However, valuable information is lost using this approach, such as how stable the MAX phase is and which competing phases are being most competitive at a given M_*n*+1_AX_*n*_ composition. This is solved by excluding the MAX phase in focus from the set of competing phases, and the solution which accounts for this is to evaluate the formation enthalpy, defined as
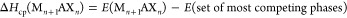
2where *E*(M_*n*+1_AX_*n*_) is the calculated total energy of an M_*n*+1_AX_*n*_ phase and *E*(set of most competing phases) is a linear combination of the identified set of most competing phases at the M_*n*+1_AX_*n*_ composition. A M_*n*+1_AX_*n*_ phase is considered stable if Δ*H*_cp_ < 0 and it is not stable, or at best metastable, if Δ*H*_cp_ > 0. This approach has been proven successful to confirm the stability of already synthesized MAX phases as well as predicting the stability of new ones.^127,129,131−136^ This approach also gives valuable information about the most competing phases with respect to M_*n*+1_AX_*n*_, knowledge that could be used for designing synthesis attempts to avoid the formation of unwanted secondary phases.

The fact that theoretical stability predictions of MAX phases reflect what has been reported experimentally, despite being performed under constraints of 0 K, can be related to mutual cancellation of temperature-dependent terms, like electronic entropy and vibrational entropy (phonons), of the MAX phases and their competing phases.^137,138^

**Figure 5:**
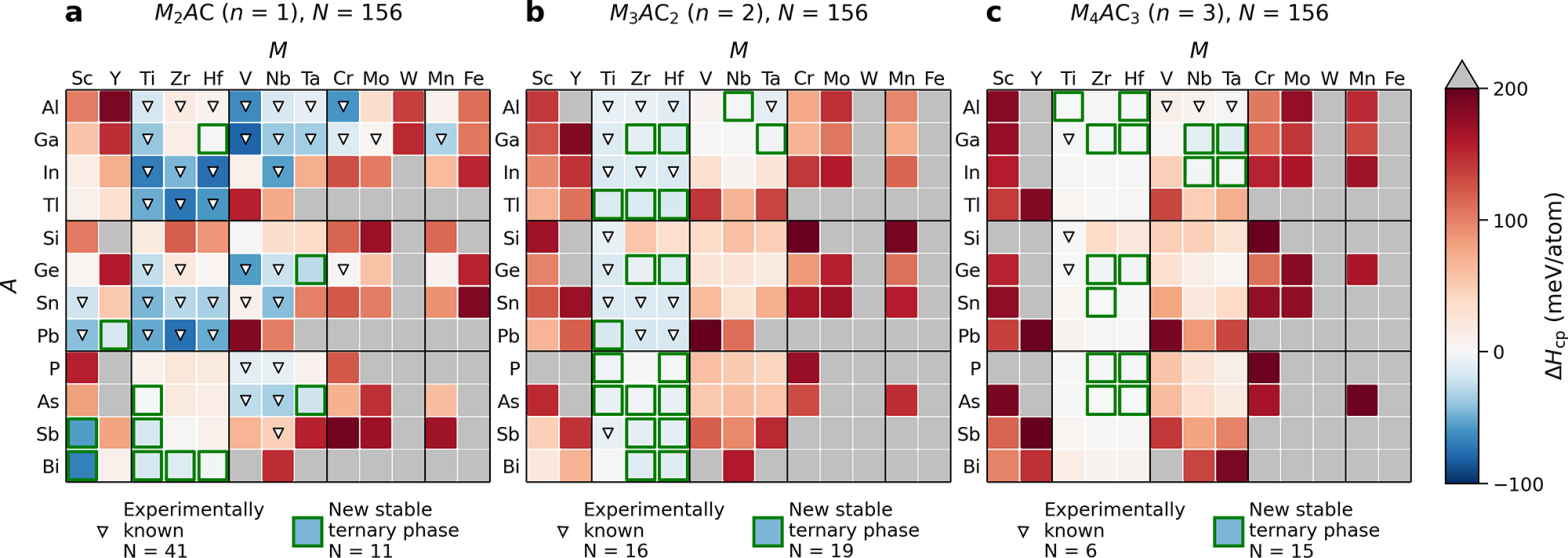
Calculated phase stability for C-based MAX phases, for (a) 211, (b) 312, and (c) 413 compositions. Triangles mark already synthesized MAX phases, and green squares mark yet to be synthesized MAX phases that are predicted to be stable with Δ*H*_cp_ < 0. Blue color represents stable phases with Δ*H*_cp_ < 0, red color represents phases with 0 < Δ*H*_cp_ < +200 meV/atom, and gray represents compositions far from stable with Δ*H*_cp_ > +200 meV/atom. (a–c) Reproduced with permission from ref (24).

Figure 5 shows results from a recent theoretical stability study of 468 M_*n+*1_AX_*n*_ phases where 98 were found to be stable with Δ*H*_cp_ < 0.^24^ Of these, 53 have been synthesized. The other 10 carbide MAX phases synthesized were found to be close to stable with 0 < Δ*H*_cp_ < 15 meV/atom. Also note the difference in stability of M_2_AX, blue regions, compared to M_3_AX_2_ and M_4_AX_3_, where another 211 phases were found to be stable. This can be related to higher-order MAX phases almost always having a M_2_AX phase among its competing phases,^24^ which also may explain the many M_2_AX phases synthesized to date and the challenges faced when targeting synthesis of M_3_AX_2_ and M_4_AX_3_.

It should be noted that positive formation enthalpies, i.e., Δ*H*_cp_ > 0, have been reported for synthesized phases, and such enthalpies should therefore not necessarily exclude synthesis attempts. Notably, approximately 20% of the materials in the Inorganic Crystal Structure Database have been reported with formation enthalpies larger than 36 meV/atom.^139,140^ In a similar comparison for 96 synthesized ternary MAX phases, 11% were found with Δ*H*_cp_ > 25 meV/atom.^24^ Part of the explanation of these diverging results, i.e., synthesis of a material predicted to be unstable, lies in the fact that phase stability is typically evaluated for an assumed ideal structure where each site is fully occupied by a single element, thus neglecting influence from vacancies and other defects that may alter the calculated energies, not only for the MAX phases but also for the competing phases. Maybe the most highlighting example is Ti_2_AlC, also converted to MXene, which typically has a C occupancy of 0.8.^141^ Another example is the 413 MAX phases Ta_4_AlC_3_ and Nb_4_AlC_3_, where ordered C-vacancies have been reported experimentally,^53,142^ which claimed to make the MAX phase more stable.^143^

Note that predicting stability for MAX phases through the use of formation enthalpy Δ*H*_cp_, eq 2, requires a complete set of competing phases. Neglecting or missing low energy competing phases will result in an overestimated stability of the MAX phase, as demonstrated in the literature,^144^ where a known Fe_3_AlC perovskite was left out when evaluating the stability for the Fe_2_AlC MAX phase, resulting in Δ*H*_cp_ = −180 meV/atom. This can be compared to Δ*H*_cp_ = +116 meV/atom when including Fe_3_AlC as a competing phase.^145^ Excluding other MAX phase compositions when identifying the set of most competing phases will also lead to an overestimated stability, which is particularly important when predicting the stability of higher-order MAX phases, *n* > 1.^119,146^

**Figure 6:**
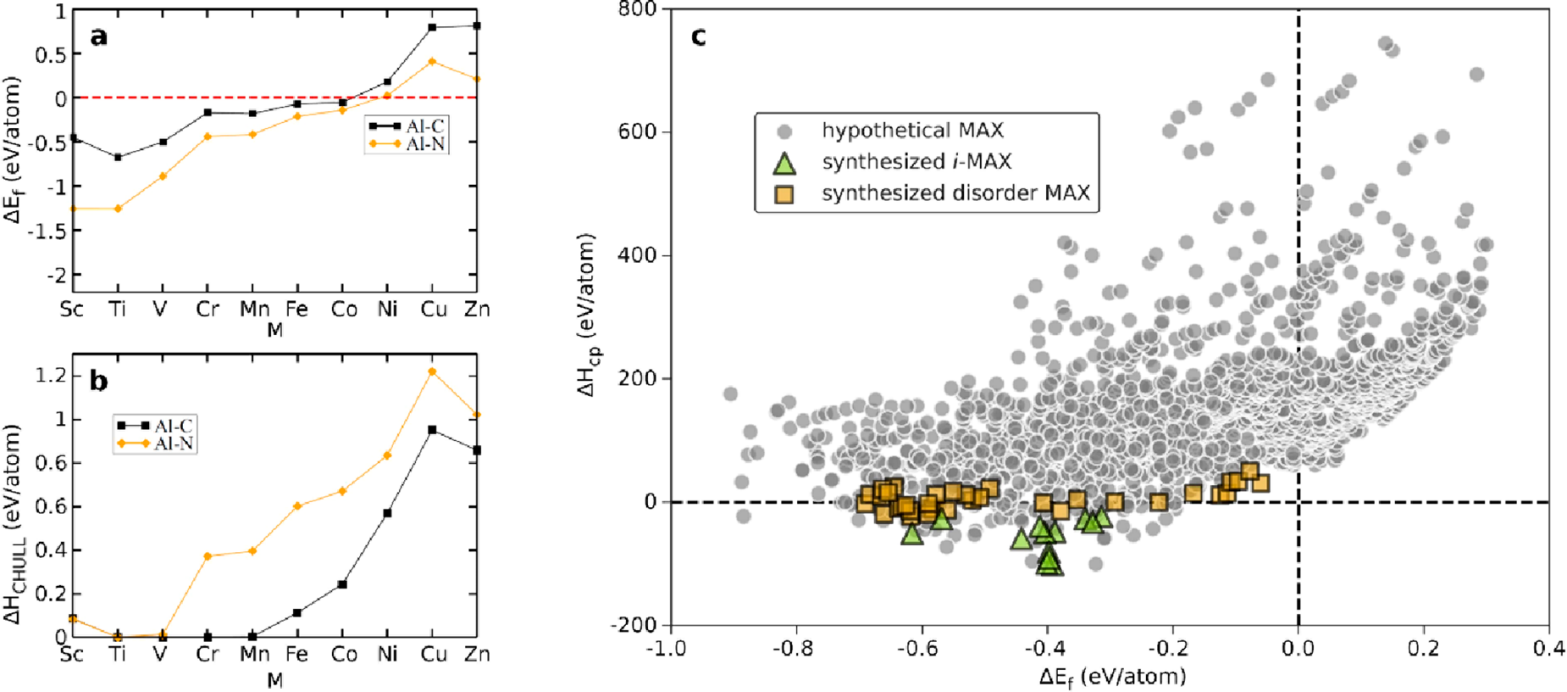
Comparison of (a) formation energies Δ*E*_f_ and (b) corresponding distance to the convex hull, Δ*H*_CHULL_, for M_2_AlC and M_2_AlN compounds. Despite the smaller formation energy, more nitrides are above the convex hull, i.e., Δ*H*_CHULL_ > 0, compared to the carbides. (c) Calculated formation enthalpy Δ*H*_cp_ as a function of formation energy Δ*E*_f_ for M′_4/3_M′′_2/3_AC *i*-MAX phases. Experimentally reported *i*-MAX phases are represented by green triangles, and compositions with a reported solid solution are represented by orange squares. Hypothetical compositions are indicated by gray circles. (a–c) Reproduced with permission from refs (127 and 147). Copyright 2019 American Physical Society and 2022 Royal Society of Chemistry, respectively.

Figure 6 shows a comparison of evaluated phase stability based on formation energy and formation enthalpy, Δ*H*_cp_. The figure clearly demonstrates that the former is a poor indicator for stability, only accounting for the decomposition of a MAX phase with respect to elemental phases. Comparing this to the formation enthalpy Δ*H*_cp_, it does reflect synthesized MAX phases quite well when the possibility for decomposition into competing phases is considered. In Figure 6a, it is found that Δ*E*_f_ for M_2_AlN is consistently lower than M_2_AlC, at a given M. This can be compared with the calculated stability in Figure 6b, represented by Δ*H*_CHULL_, for which M_2_AlC is more stable compared to M_2_AN,^127^ though only predicted to be experimentally attainable for Δ*H*_CHULL_ = 0. The latter reflects previously synthesized MAX phases, where three carbides (Ti_2_AlC, V_2_AlC, and Cr_2_AlC) and only one nitride (Ti_2_AlN) have been reported. Another example is given in Figure 6c where the formation enthalpy Δ*H*_cp_ is compared to the formation energy Δ*E*_f_ for quaternary M_2_AX phases, with synthesized compositions highlighted.^147^ It is clear that Δ*E*_f_ provides no valuable information from a stability point of view, and using this value as guidance may lead to erroneous conclusions and wasted experimental efforts.

##### Elemental Mixing in Alloys—Impact from Chemical Order and Temperature

Moving beyond ternary MAX phases means that we have to add additional elements on the M, A, or X site. There are two important aspects when performing an evaluation of phase stability of MAX phase alloys, e.g., double-metal MAX phases. The first is how the alloying elements are distributed. For a long time, only solid solution (chemical disorder) was known in MAX phases. Models approximating such disordered distributions are constructed by, for example, creating random structures using the special quasi-random structure (SQS) method, which compares correlation functions of a finite unit cell to those of an infinite ideally random system.^148^ The idea with the SQS method is to minimize the difference in the correlation functions between the modeled cell and the ideal system, and it is considered to give a good approximation for describing near-randomness in solid solution alloys. This has been demonstrated for various systems, including MAX phases.^147,149^

**Figure 7:**
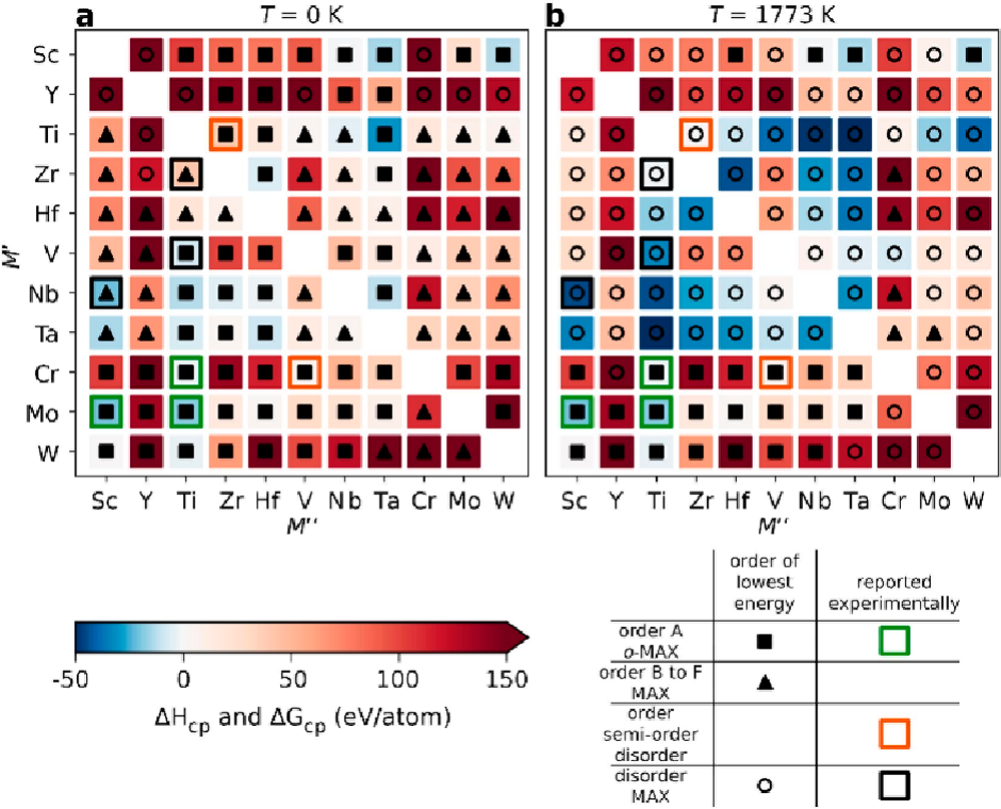
(a–b) Calculated stability for 312 MAX phase structures with a 2:1:1:2 composition of M′/M′′/Al/C, indicating if chemical order (filled squares for *o*-MAX and filled triangles for order B to F) or disorder (open circles) is preferred at (a) 0 K and (b) a typical synthesis temperature of 1773 K. In addition, experimentally reported phases are marked by open squares where their color indicates reported order; green (*o*-MAX), orange (*o*-MAX, semiorder, or solid solution), and black (solid solution MAX). (a–b) Reproduced with permission from ref (150). Copyright 2020 Royal Society of Chemistry.

However, with the discovery of out-of-plane order for Cr_2_TiAlC_2_*o*-MAX^95^ and in-plane order for Mo_4/3_Sc_2/3_AlC *i*-MAX,^84^ it is necessary to also consider chemical order in the MAX phase studied, since it is challenging to *a priori* know the distribution of alloying elements to be expected upon synthesis. Another aspect which has been demonstrated to be critical when studying MAX phase alloys is to also account for configurational entropy when modeling disorder. Figure 7 shows the calculated stability for 312 MAX phase alloys considering both solid solution (chemical disorder) and out-of-plane (*o*-MAX) order, where the impact of configurational entropy is considered (panel b) or not (panel a).^150^ At 0 K, synthesized solid solution MAX phases (for alloying on the metal site) are neither found stable nor do they have a chemically disordered distribution of lowest energy. However, when accounting for configurational entropy at 1773 K, these phases become stable, with a disordered configuration of lowest energy.

Predicting stable MAX phases through theory has guided subsequent synthesis of novel single-metal MAX phases,^131,133^ double-metal MAX phases with a random arrangement of M′ and M′′,^62,67,69,132,134,151,152^ and most recently high-entropy MAX phases.^73^ Multiple chemically ordered MAX phases have also been found through theoretical guidance and include both out-of-plane *o*-MAX^93,98^ and in-plane ordered *i*-MAX.^85,88,111,112,153^ Performing reliable theoretical studies to be used as guidance for future synthesis of novel MAX phases therefore requires consideration of complete sets of competing phases and also ordered and disordered atomic configurations when mixing elements, including consideration of configurational entropy.

#### 2.2.3 Machine Learning Predictions

While high-throughput phase stability studies of MAX phases are a powerful tool for the identification of stable materials, it is a quite demanding approach.^127,136,147,150,154^ Using machine learning (ML) for accelerating predictions of the synthesizability of MAX phases has emerged as a tempting approach. Frey et al. used positive and unlabeled ML to predict those MAX phases having the highest likelihood of being synthesized using a feature set composed of elemental data (e.g., atomic number, group number, ionization potentials, etc.), DFT calculated features related to structure (number of layers, lattice constants, layer distances, bond lengths, etc.), and thermodynamic data, including calculated total energy, formation energy, and cohesive energy.^155^ They found that the top five most important features were the formation energy, number of layers, Bader charge of M atom, mass of MAX phases, and cohesive energy. This indicates that this ML model is highly dependent on the thermodynamic calculated data considered in the study, which for this case are formation energy and cohesive energy. Using the ML model, they accounted for 792 MAX phase compositions and predicted 111 promising candidates with a high synthesizability score.^155^ It is important to note that since formation energy was used and identified as the most important feature descriptor, it is expected that the synthesizability is highly overestimated, as discussed in section 2.2.1. A closer look at the 111 candidate MAX phases,^155^ for which calculated formation enthalpies can be found in the literature,^24,146^ reveals that many of the compositions are far from stable. This is a consequence of using formation energy as a feature descriptor in the ML model.

## 3 Computationally Driven MXene Development

There has been a vast number of theoretical studies involving MXenes in one way or another. This has partly been motivated by the large number of existing MAX phases in combination with the relatively small proportion of these actually being utilized for chemical exfoliation, i.e., it is natural to imagine a plethora of MXenes waiting to be discovered. By combining different M and X elements and various surface terminations, hypothesized structures of 23,870 unique MXenes have been generated.^156^ As it appears that a virtually endless number of MXenes is possible, a tempting approach for theoreticians is to predict which (most probably hypothetical) MXene has the most promising properties toward a particular application, leaving its realization to experimentalists. However, we also need to consider if and how such MXenes could be synthesized. If following the conventional top-down chemical exfoliation, we need a suitable parent MAX phase that is thermodynamically stable, as discussed in section 2. We also need to assess whether the MAX phase is chemically exfoliable, and it is also necessary to consider different etching protocols (e.g., acid vs molten salt). The reliance of such predictions depends on how well we understand the underlying mechanisms governing the etching. The goal of this section is to review the available literature on theoretical studies aiming at predicting or understanding the top-down synthesis of MXenes from parent MAX phases. As will be shown, depending on underlying assumptions, it is possible to reach different conclusions regarding the synthesizability of MXenes. Therefore, accurate assumptions with the perception of reality are a prerequisite.

### 3.1 Multilayer MXenes and Delaminated Freestanding 2D MXene Sheets

The first question we need to resolve, before entering into a discussion of how the synthesis of 2D MXenes can be predicted, is what constitutes a 2D material. A strict mathematical definition would be a material with units periodically repeating in 2D, while it is atomically flat perpendicular to these two directions. This view is heavily influenced by the archetype of any 2D material, namely, graphene. However, the ideal view of a completely flat material is an approximation even in the case of graphene, particularly when considering its flexibility and vibrational modes. And even if the nuclei are approximated as point-masses perfectly constrained in a 2D landscape, the electronic structure extends well outside this plane. Thus, the definition of a physical 2D material should rather be considered as a structure with periodic extension in 2D but allowed to have, to some extent, structural components outside the 2D plane. Typically, this includes materials with up to a few atomic layers, although there is no clear consensus on this topic.

In the world of MAX phases and MXenes, the question of what constitutes 2D is taken to its extreme. A parent MAX phase consists of a MXene substructure and an A layer. But the MAX phase is a complete 3D layered material. During what part of the process from MAX to MXene do we go from 3D to 2D? The question may seem semantical but is important to allow the comparison of MXenes to other 2D materials.

If restricting ourselves to top-down fabrication of MXenes, the successful synthesis requires the removal of the A element. However, there is no one-to-one relation between the removal of the A element and the formation of a MXene. For example, an early study on the Ti_3_SiC_2_ MAX phase illustrated the possibility of removing the Si from the material by annealing to sufficiently high temperatures.^157^ However, this does not result in a layered material consisting of separated MXenes, but instead the material shrinks in the direction perpendicular to the MXene planes and collapses into a reconstructed bulk phase. There are also examples of materials with only partial etching of the A layer from MAB phases,^158−161^ closely related to MAX phases, which is clearly not sufficient to comply with successful exfoliation, i.e., for the selective etching to be considered successful in relation to MXene synthesis, the A layer needs to be completely removed while avoiding the etched structure collapsing into a new bulk structure, i.e., the etched MAX phase should consist of chemically inert MXene substructures, not connected by chemical bonding.

However, although a MAX phase is successfully etched according to the criteria above, the as-produced MXene is typically in its multilayer form, where weak interactions similar to van der Waals forces still exist between the layers. Further delamination steps are needed to separate the multilayer MXenes into freestanding 2D sheets. For example, Kamysbayev et al. demonstrated the possibility of etching MAX phases in CdBr_2_ molten salt, resulting in successful etching of the A element and resulting MXenes terminated by Br (which could subsequently be replaced by other terminations or removed).^7^ However, it should be noted that the resulting structures were multilayer MXenes with van der Waals-like interactions between the layers, i.e., a MXene was produced, but without delamination freestanding 2D sheets are not obtained. If a distinction between multilayer MXene and corresponding single sheets are not made, any layered material stabilized by nonchemical interactions between the layers would be considered as 2D materials and, e.g., the mechanical exfoliation of graphite^162^ would not have been such a great deal.

Before presenting the different efforts made to understand the synthesis, it is necessary to say a few words about surface terminations. The removal of the A elements results in undercoordinated M elements being exposed. This inevitably leads to the bonding of termination groups to the MXene surfaces during the etching, where the type of terminations is determined by the etching conditions. While close to nonterminated MXene has been shown experimentally, this is limited to either high-vacuum conditions or multilayered MXenes not exposed to the atmosphere.^7,163^ As the surface terminations form during the selective etching, realistic theoretical descriptions of MXene synthesis presumably take these into consideration.

### 3.2 MXene Stability Compared to Different Elemental Reference Phases

One strategy that has been used for predicting if a MXene can be synthesized or not is to consider the formation energy with respect to different references for the chemical elements. Generally, the formation free energy of a MXene can be defined as
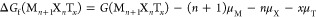
3where *G*(M_*n*+1_X_*n*_T_*x*_) is the free energy of the MXene with surface terminations T_*x*_, while μ_M_ and μ_X_ give the chemical potentials of the M and X elements, respectively, and μ_T_ is the chemical potential of the termination species. In the majority of studies, the different terms are calculated from the total energy at 0 K, obtained from electronic structure theory calculations, and eq 3 is simplified to a formation enthalpy, which we will refer to as the formation energy. A negative formation energy simply tells us that the MXene is more stable than the elemental reference phases used to construct the chemical potentials.

By defining the chemical potentials as the energy of the elemental reference phases, the formation energy of the Sc_2_C MXene without terminations (*x* = 0) has been calculated to be −0.144 eV/atom, reaching the conclusion “*··· which shows that it is stable and can be synthesized”*.^164^ In spite of the fact that 2D Sc_2_C was predicted to be synthesizable almost ten years ago, and with the additional incentive that this would be the lightest MXene with the highest surface area per weight, meaning an outstanding hydrogen storage capacity, it has not to date been synthesized by chemical exfoliation of a MAX phase.

**Figure 8:**
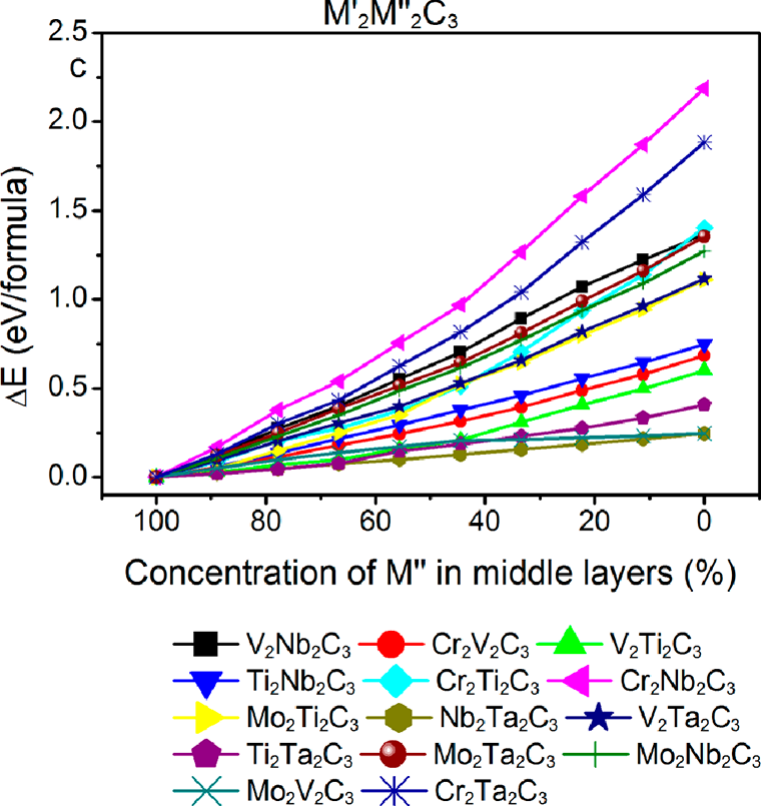
Computed relative stability of M′_2_M′′_2_C_3_ MXenes for different combinations of M′ and M′′, as a function of the fraction of M′′ in the middle layers. In all considered combinations, structures with chemical out-of-plane order are energetically preferred over disordered structures, if choosing the correct M elements are put in the middle and outer layers, respectively. Reproduced with permission from ref (96). Copyright 2015 American Chemical Society.

The stability of MXenes is not always evaluated by the formation energy. For example, MXenes containing two transition metal elements M′ and M′′ with the chemical formulas M′_2_M′′C_2_ and M′_2_M′′_2_C_3_ were studied by comparing the relative energies of MXenes with the same chemical composition.^96^ The aim was to identify how out-of-plane chemical ordering between the two elements affects the relative stability when combining two of the M elements Ti, V, Nb, Ta, Cr, or Mo.^96^ It should be noted that the obtained results do not necessarily reflect the potential for experimental realization but rather if different combinations of transition metals prefer to be combined in chemically out-of-plane ordered or disordered fashion into MXene structures. Furthermore, the effect of surface terminations was not considered. For M′_2_M′′C_2_ MXenes, out-of-plane chemical ordering is obtained if M′ atoms are placed in the two outer planes and M′′ is in the inner plane of the MXene, while for M′_2_M′′_2_C_3_ this is achieved if all M′ (or M′′) atoms are placed in the outer plane and the other element is in the two inner planes. For the M′_2_M′′_2_C_3_ stoichiometry, the out-of-plane ordering was preferred for all tested combinations, as long as selecting the right transition metal in the outer layer and the right one in the inner layer, as shown for a selection of M′–M′′ combinations in Figure 8. Among all the considered M elements, Mo and Cr have strongest preference to occupy the outer plane, while Ta has the strongest preference to sit in the inner plane. In the case of M′_2_M′′C_2_, whether chemical ordering is preferred or not depends on the combination of M′ and M′′. While the study did not evaluate the thermodynamic phase stability, 20 previously unknown *o*-MXenes were suggested, of which Mo_2_TiC_2_, Cr_2_TiC_2_, and Mo_2_Ti_2_C_3_ were experimentally verified. However, following Table 1, among the predicted 20 *o*-MXenes, the only additional one out of these which has been synthesized is Mo_2_Nb_2_C_3_^98^ (and additionally one *o*-MXene with Sc,^93^ but this was not part of the materials considered in this study). It should be noted that the two MAX phases Cr_2_VAlC_2_ and Cr_2_V_2_AlC_3_ were realized already in 2015,^108^ and although the corresponding MXenes Cr_2_VC_2_ and Cr_2_V_2_C_3_ are in the list of predicted *o*-MXenes,^96^ they have not been realized to date. This highlights the fact that the relative energies between MXenes have little to say about their synthesizability.

**Figure 9:**
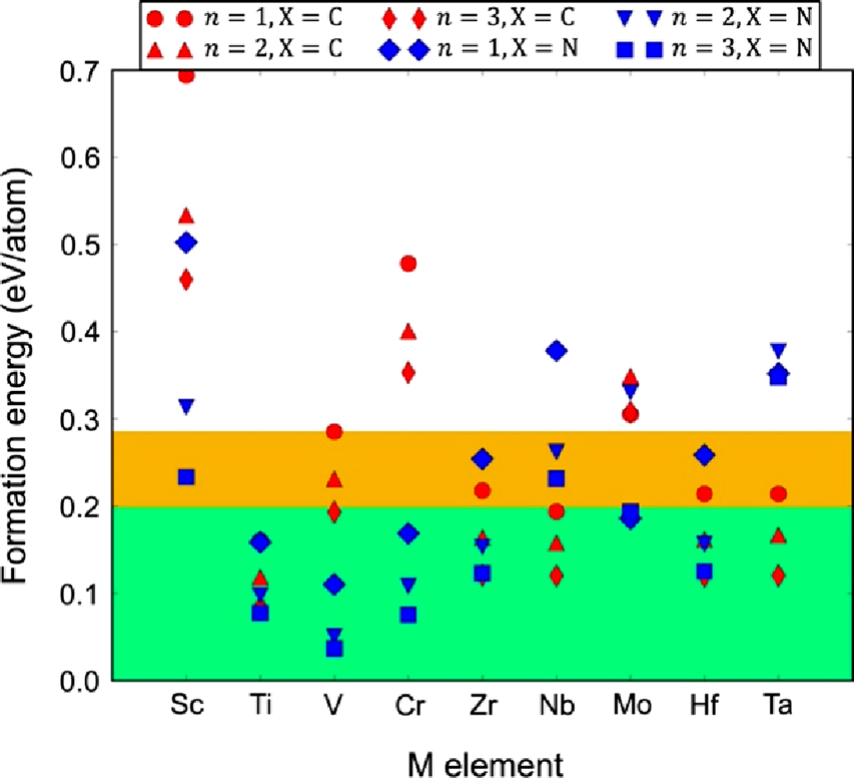
Formation energies for M_*n*+1_X_*n*_O_2_ MXenes (i.e., fully terminated by O) with respect to the lowest energy mixture of competing bulk phases. The green region highlights the phenomenological threshold for existing 2D materials of 0.2 eV/atom,^165^ and the yellow region highlights the 0.285 eV/atom formation energy of the V_2_CO_2_ MXene, the highest of the considered MXenes that have been experimentally synthesized. Reproduced with permission from ref (165). Copyright 2016 American Chemical Society.

The studies we have mentioned so far are all based on total energies of the MXenes compared to different reference values (either elemental phases or other MXenes). As concluded in section 2, a comparison to elemental phases for evaluation of phase stability can be misleading, as it does not take into account competing phases that are potentially more stable than the considered material. Notably, a study by Ashton et al. calculated the formation energies with respect to competing bulk phases of 54 MXenes (by combining different transition metals with C or N for different numbers of layers), concluding that no MXenes are thermodynamically stable,^165^ as shown in Figure 9, i.e., the energy with respect to competing phases is positive, which is a general property for any 2D material.^166^ At best, MXenes are metastable, implying that, if kinetically accessible, they will decompose into more stable phases. Notably, from the trend of these formation energies, there is no clear correlation with respect to which MXenes have been experimentally verified. The studies presented so far have one thing in common: the fact that MXenes are typically synthesized from parent MAX phases is neglected.

### 3.3 MXenes from MAX Phases

To understand the synthesis of MXenes from parent MAX phases, and being able to computationally predict new MXenes created this way, the computational models need to take into consideration the process of going from MAX phase to MXene, i.e., modeling a chemical reaction with the general equation
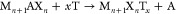
4in which the A element is selectively etched from M_*n*+1_AX_*n*_ to form the M_*n*+1_X_*n*_T_*x*_ MXene. This equation also takes into account the formation of terminations T on the surface of the MXene. Based on this, a general exfoliation (free) energy can be defined as
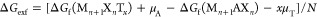
5where Δ*G*_f_(M_*n*+1_AX_*n*_) and Δ*G*_f_(M_*n*+1_X_*n*_T_*x*_) are the formation free energies of the MAX and MXene, respectively. Notably, the energies of MAX and MXene are usually approximated by the corresponding total energies at 0 K obtained by electronic structure theory calculations. The chemical potentials μ_A_ and μ_T_ can be viewed as reference energies of the A element and the termination species. Depending on how these chemical potentials are defined, eq 5 can be viewed as a free energy or as an enthalpy (normally referred to as an “energy” by the Condensed Matter Physics Society). When discussing exfoliation, we will use the general term *exfoliation energy* but indicate how the chemical potentials were treated. The factor *N* is a normalization factor which can be defined in different ways depending on the desired unit of the exfoliation free energy. For example, it may be defined as the area of the MXene units^167^ or by the number of atoms in the MAX precursor.^168^ It is important to have in mind using the same normalization factor for competing processes, such as the complete solvation of the material (*vide infra*).

#### 3.3.1 Mechanical Exfoliation

A simplified way of applying eq 5, used in a number of studies, is assuming the A element in their elemental reference phase (for which μ_A_ = 0) and ignoring the formation of termination groups (*x* = 0). Under such assumptions, eq 5 takes the specific form
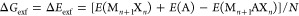
6where *E*(M_*n*+1_AX_*n*_), *E*(M_*n*+1_X_*n*_), and *E*(A) are the total energies (at 0 K) of the MAX phase, the MXene, and the A element in its elemental crystal phase, respectively. This treatment of exfoliation can be seen as a mechanical exfoliation in which the MXene layers are pulled from each other, leaving the A layer behind, and does not account for the chemical processes taking place in a real system. (Note, not all studies acknowledge this as mechanical exfoliation, although we describe them as such in this Review.)

An early study, treating the MXene synthesis as a mechanical exfoliation, investigated how the choice of A element affects the exfoliation of Mo_2_AC MAX phases.^169^ Around the same time, the approach was also used for studying the formation of a range of MXenes from different MAX phases.^170^ Both studies demonstrated that the mechanical exfoliation is significantly endothermic for all MAX phases. Notably, among the 42 MAX phases considered in the latter study, Sc_2_AlC was considered to be the most easily exfoliated one.^170^ As already mentioned, the Sc_2_C MXene has to date not been realized by chemical exfoliation.

**Figure 10:**
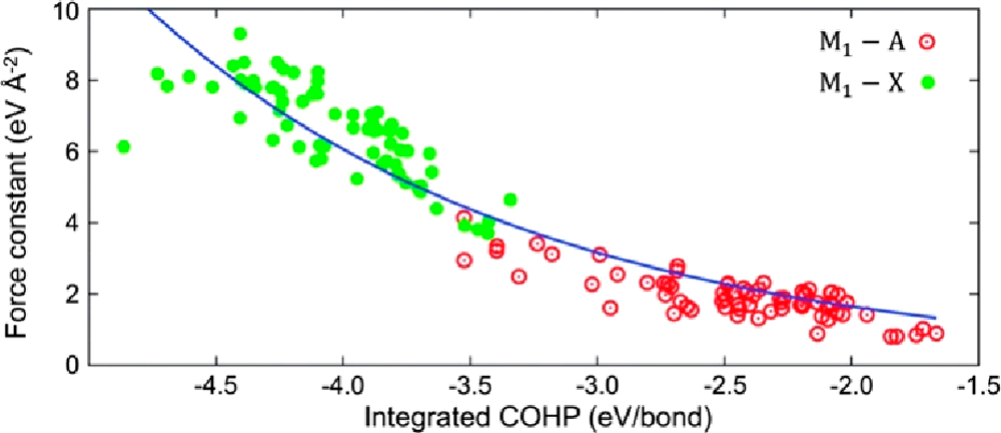
Bond strength versus force constant for the M_1_–A and M_1_–X bonds for various experimentally synthesized MAX phases. Here the bond strength is quantified by the integrated crystal orbital Hamilton population (COHP) up to the Fermi energy over all the atomic orbital interactions between the atoms forming the bonds. The line is a guide to the eye. Reproduced with permission from ref (169). Copyright 2014 IOP Publishing.

Later, a similar approach was used for calculating exfoliation energies under the same assumption of mechanical exfoliation, i.e., with *x* = 0 and μ_A_ = 0, but using a different normalization factor.^167^ The study considered 82 experimentally synthesized MAX phases and corroborated the earlier results that Sc_2_AlC has the lowest exfoliation energy. In fact, none of the 13 MAX phases with the lowest exfoliation energies in this study have been selectively etched experimentally. Based on these results, first of all, it is challenging to understand why MAX phases can be exfoliated into MXenes at all, considering that exfoliation is considered endothermic independent of the choice of MAX phase. Second, not even the trends of the exfoliation energies can be correlated with what is known from experimental work. It should be noted that in this study the analysis also considered force constants of M–X and M–A bonds as well as chemical bond analysis with so-called crystal orbital Hamilton population (COHP). As shown in Figure 10, this analysis shows that both the force constants and chemical bonding are stronger for M–X bonds than M–A bonds, qualitatively explaining why M–A bonds are more easily dissociated than M–X bonds, which one may intuitively expect for selective etching of the A elements. Using experimentally exfoliated MAX phases, limits were put on bond strength and exfoliation energy for a MAX phase to be predicted as synthesizable. If a MAX phase is found within all three limits, it is considered to be exfoliable. The analysis resulted in a range of MAX phases predicted to be possible to convert into 2D, such as the formation of Cr_2_N, Cr_2_C, and Zr_2_C MXenes, not reported to date.

Following a similar approach as in the literature,^167^ a large-scale study was performed attempting to computationally predict new MXenes by considering a larger collection of possible candidates.^146^ First, it was studied which MAX phases are expected to be synthesizable. Based on the exfoliation energy for mechanical exfoliation (*x* = 0 and μ_A_ = 0) and force constants, MXenes were predicted. As in the literature,^167^ the criteria for acceptable values of exfoliation energies and force constants were defined by MAX phases exfoliated experimentally. The same strategy was also used in another study, which also correlated the mechanical exfoliation energy (*x* = 0 and μ_A_ = 0) with the adsorption energy of CO_2_ with nonobvious linear correlations.^171^

It is clear from these initial studies, using the exfoliation energy alone through the (often implicit) assumption of mechanical exfoliation, that without a relevant treatment of the chemical environment through the chemical potentials μ_A_ and μ_T_, it is not possible to give a coherent explanation of which MAX phases can be exfoliated or not. In particular, this is evident as all the studies presented in this section imply that it is thermodynamically unfavorable to exfoliate a MAX phase into a MXene. If a methodology does not describe the exfoliation as a thermodynamically favorable process for experimentally known synthesis protocols, it is questionable how relevant it is for predicting new MXenes. Notably, other relevant information may be extracted from such studies, though related neither to stability nor exfoliability.

#### 3.3.2 Electrochemical Exfoliation and Pourbaix Diagrams

**Figure 11:**
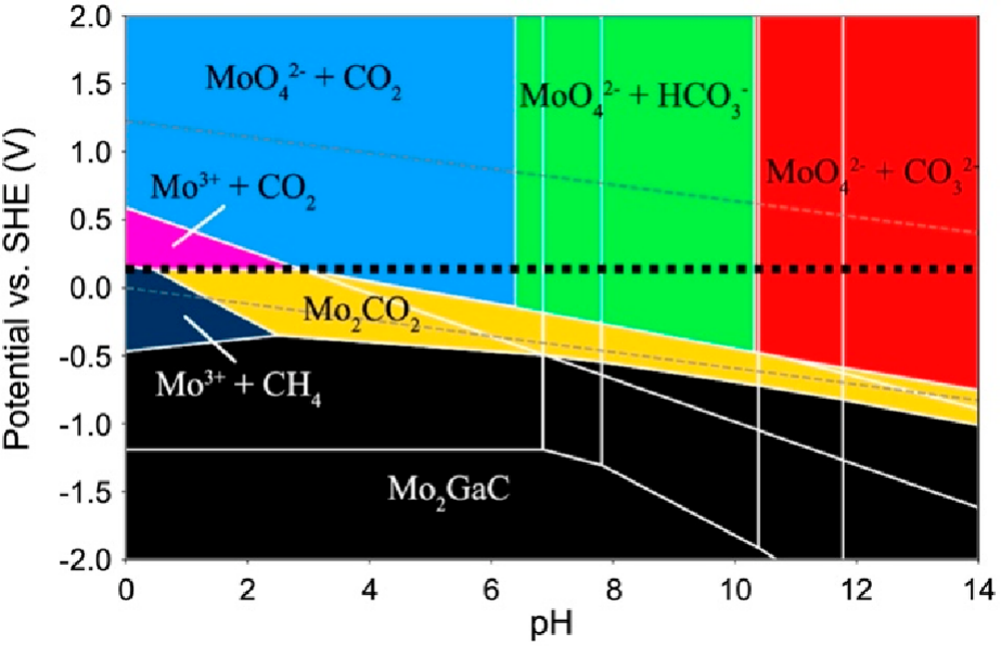
Chemical etching diagram (Pourbaix diagram) demonstrating the region of thermodynamical stability of the Mo_2_GaC MAX phase and Mo_2_CO_2_ MXene as a function of pH and electrode potential. Reproduced with permission from ref (172). Copyright 2019 American Chemical Society.

As concluded in previous section, without an accurate description of the chemical environment, it is difficult to make predictions of chemical exfoliability. Ashton et al. made an important contribution toward studying the exfoliation of MAX phases under realistic conditions by considering the particular case of electrochemical etching.^172^ In their study, the exfoliation energy was not explicitly calculated, but instead Pourbaix diagrams were considered, which shows under which pH values and electrode potentials MXenes and MAX phases are more stable than their respective dissolved species. The Pourbaix diagrams were constructed by only including the respective MAX and MXenes as allowed solids, and all molecules and ions including any of the elements of the MXene, as well as H, O, and F, mimicking a situation where the electrochemical etching takes place in HF. Formation energies of MAX phases and MXenes were computed with DFT, while for dissolved species experimental formation energies from databases were used. The Pourbaix diagrams were used to decide whether the MAX phase, the MXene, or a combination of dissolved molecules and ions is thermodynamically most stable at a given combination of pH and electrode potential. In Figure 11, a typical result is exemplified by a Pourbaix diagram for the electrochemical etching of Mo_2_GaC. The yellow area indicates the electrochemical conditions at which it is predicted that the Mo_2_CO_2_ MXene (Mo_2_C with O termination) is more stable than the Mo_2_GaC MAX phase as well as all competing dissolved species. This implies that the formation free energy in eq 5 is negative when applying an electrochemical treatment of the chemical potentials μ_A_ and μ_T_. The Pourbaix diagram also implies under what conditions the Mo_2_GaC MAX phase is more stable than all competing phases, indicating both a positive formation free energy and a resilience against solvation. It is important to note that Mo_2_GaC has not been successfully etched to date. However, for Mo_2_Ga_2_C (which differs from conventional MAX phases by its double A layer, see Figure 3a) the same study showed theoretically that the MXene synthesis is extended over a wider area of pH and electrode potential. This trend is in qualitative agreement with experiments, as Mo_2_Ga_2_C is experimentally exfoliable. Notably, conventional MXenes are not synthesized electrochemically, and it would be interesting to investigate how accurate the predictions of ideal electrochemical etching conditions are. Furthermore, such Pourbaix diagrams could be valuable to indicate under which conditions MXenes can be expected to be stable under electrochemical conditions. Especially, such information is instrumental when utilizing MXenes in, e.g., electrocatalysis.

The approach of constructing Pourbaix diagrams has also been used by Caffrey to study optimal (electrochemical) etching conditions to form *o*-MXenes.^173^ In this study, also the effect of different surface terminations was considered, showing an, in general, larger stability window for F-terminated MXenes than O-terminated ones, while OH-terminated ones were shown to be the generally least stable, though this was not always the case. In other words, by also including different termination scenarios, Pourbaix diagrams could be used to indicate electrochemical synthesis conditions for achieving particular surface terminations.

#### 3.3.3 Chemical Exfoliation and the Concept of Electroneutrality

We have seen various examples of how to computationally predict MXenes. As MXenes generally originate from parent MAX phases, it is necessary to have a relevant representation of the chemical processes involved. Many studies have simply treated the removed A elements in their respective elemental phases and have neglected the formation of surface terminations. In the previous section, it was demonstrated how Pourbaix diagrams can be used to elucidate ideal electrochemical etching conditions. As conventional etching of MAX phases is normally not performed in an electrochemical cell, we need to consider under which conditions Pourbaix diagrams are relevant and slightly rethink to make the approach suitable for conventional etching.

The keyword is electroneutrality. In an electrochemical cell, electroneutrality is ensured by electrons being removed from the electrochemical anode where atoms dissolve into positive ions, and vice versa for the electrochemical cathode. During conventional etching we do not have the possibility to balance the electron count between two half-cells. Instead, it is necessary to impose electroneutrality on the chemical reactions. To calculate the exfoliation free energy with eq 5, the main difference is the evaluation of the chemical potentials μ_A_ and μ_T_. In both electrochemical and conventional etching, the chemical potentials are constructed for electroneutral processes. In the electrochemical case, we have an electrode potential to which we can add or remove electrons, while the conventional etching does not have the electrode potential and no possibility of adding or removing electrons.

Recently it was demonstrated how electroneutral chemical potentials can be constructed for conventional etching processes in HF,^168^ which also considered how the chemical potential of the fluorine depends on the pH of the solution. The exfoliation free energies were considered both with and without terminations, i.e., *x* = 2 and *x* = 0, respectively. Also, similar to the work on electrochemical etching, the complete solvation of the MAX phases was considered, by defining a solvation free energy as
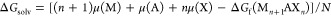
7where Δ*G*_f_(M_*n*+1_AX_*n*_) is the formation free energy of the MAX phase and μ_M_, μ_A_, and μ_X_ are the chemical potentials of the M, A, and X elements, respectively. The normalization factor *N* needs to be the same as for the exfoliation free energy in eq 5 to enable a comparison of the two processes. (In the studies of electrochemical etching, the solvation free energies were not explicitly reported but rather entered as a feature of the Pourbaix diagrams.)

Notably, making this more realistic treatment of the chemical environment, the chemical exfoliation was shown to be a thermodynamically favorable process.^168^ However, the calculations predict exfoliation to be favorable for all MXenes in the case of *x* = 2, and in the majority of cases for *x* = 0, and do not discriminate between those that can and those that cannot be etched experimentally. To understand whether an MXene can be formed or not, it is necessary to also consider competing processes. For example, the entire MAX phase might dissolve instead of forming the MXene. But it is not enough to compare solvation free energy to exfoliation free energy, as the former is more favorable with only a few exceptions, implying that the majority of MXenes will dissolve if given enough time in HF solution.

**Figure 12:**
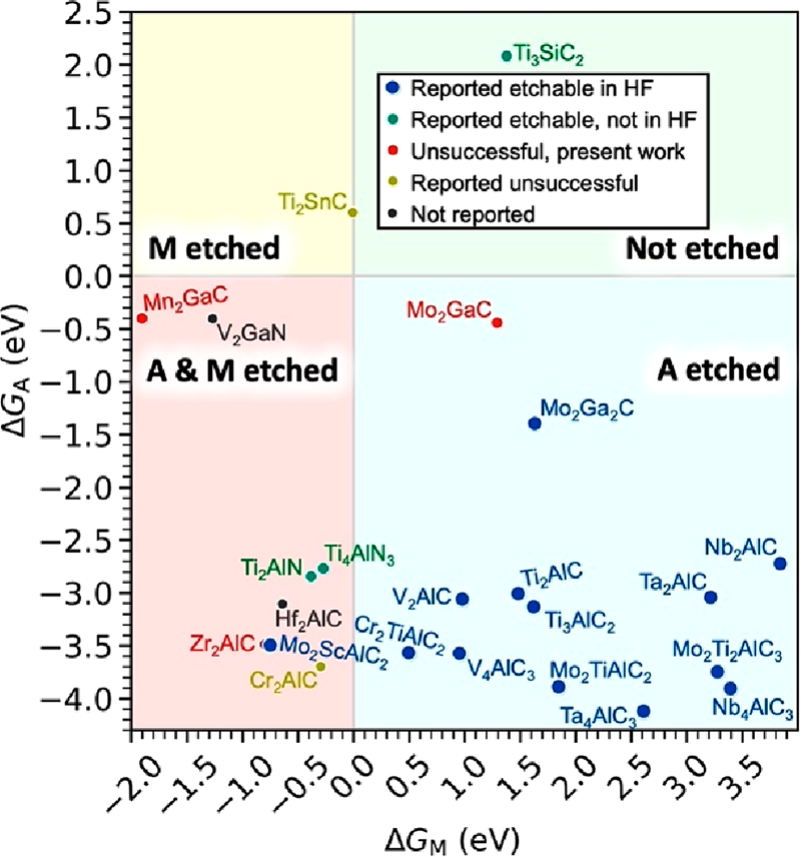
Free energy of vacancy formation of removing one A element compared to one M element atom for different MAX phases, calculated by assuming that the A and M elements dissolve into aqueous HF at pH = 0, with the restriction that oxides are not allowed to be formed under the acidic conditions. The expected etching behavior is indicated for each of the four quadrants. Adapted with permission from ref (168). Copyright 2023 Springer Nature.

Instead, the problem boils down to the thermodynamics of the initial etching process, and it is also necessary to do further modifications of the chemical potentials to better represent the chemical environment. The modification of the chemical potential was based on the notion that oxides are not likely formed under a highly acidic environment, exemplified for Si where it is well-known that an oxidant is needed to promote oxide formation in HF,^174^ i.e., all oxides were removed when constructing the chemical potentials. If considering the initial step of the etching, it was also shown that removing an A element atom is thermodynamically favorable while removal of an M element is thermodynamically unfavorable for MAX phases that are exfoliable,^168^ as demonstrated in Figure 12.

It is important to have in mind that the MXenes are predicted to dissolve in HF if given sufficient time.^168^ This is in line with the available literature and general experience of working with MXenes. Important components in the synthesis of MXenes must be that the etching of the A element precedes the solvation of the remaining material (this is undisputable and essentially the definition of selective etching) and that the solvation of the resulting MXene is sufficiently slow, such that it can be separated from the etchant solution in time. The surface terminations probably play an instrumental role here. The MXenes with surface terminations are significantly more stable than those without. To dissolve the MXene we need to remove surface terminations, which means that the solvation is somewhat a kinetically hindered process. Notably, there are indications that the degradation of MXene is initiated at the boundaries/edges,^175^ which can be slowed down by using, for example, antioxidants.^176^

**Figure 13:**
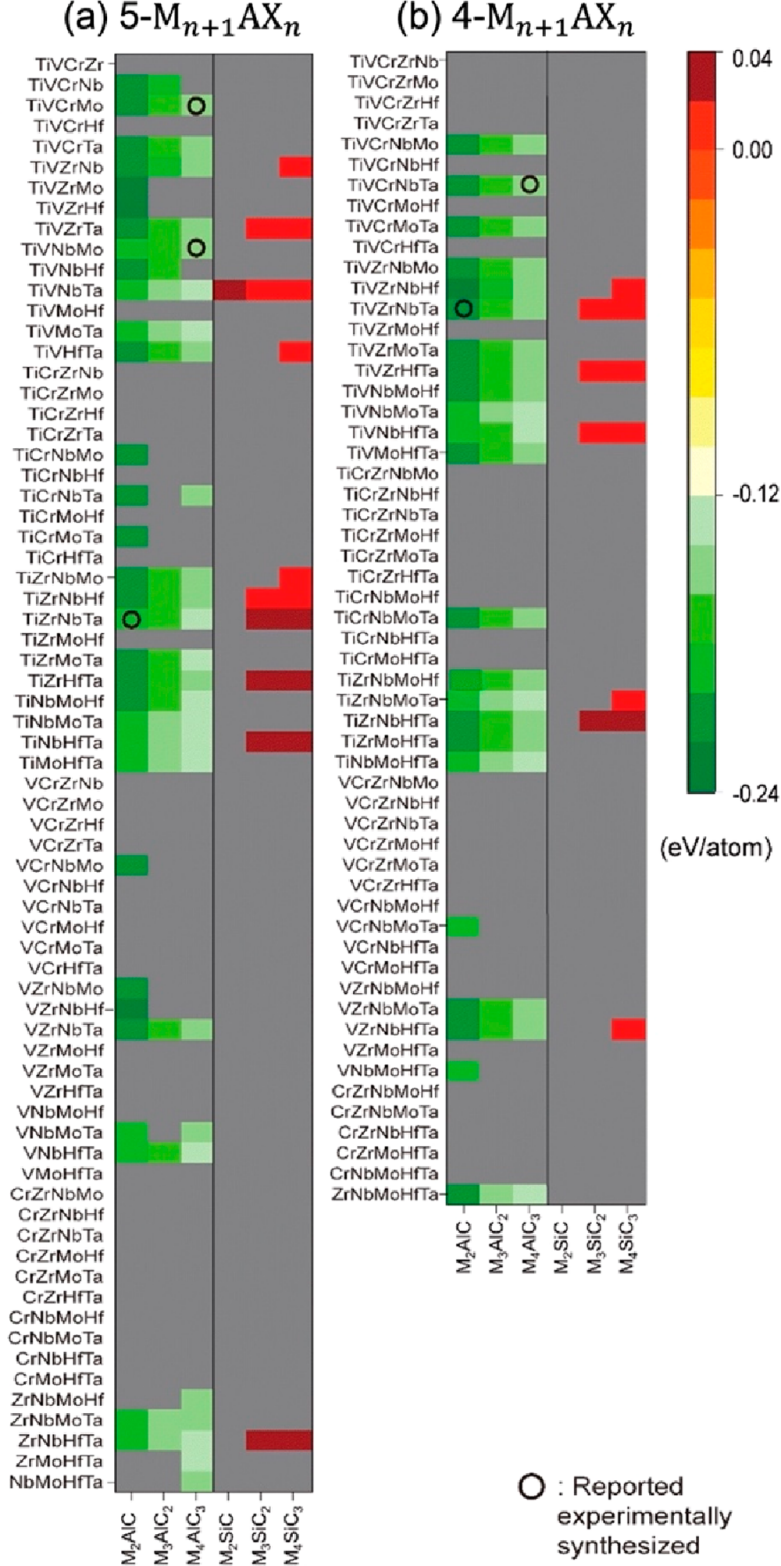
Heatmap of chemical exfoliation energy for HE-MAX phases with (a) four and (b) five different transition metals. The circle symbol represents experimentally reported HE-MAX phases. The gray color represents HE-MAX phases computed to be thermodynamically unstable. Reproduced with permission from ref (73). Copyright 2023 Royal Society of Chemistry.

The scenario of chemical exfoliation from a theoretical perspective was also used by Seong et al. to predict synthesizable high-entropy MXenes (HE-MXenes).^73^ They explicitly considered HE-MAX phases with Al or Si as the A element and combining either four or five of the transition metals Ti, V, Cr, Zr, Nb, Mo, Hf, and Ta. The chemical exfoliation was calculated using eq 5, assuming the formation of AlF_3_ and SiF_4_ and H_2_ molecules, for Al and Si, respectively, through the reaction with HF during the exfoliation. Surface terminations were not considered (*x* = 0). The study specifically considered HE-MAX phases shown to be thermodynamically stable and calculated chemical exfoliation energies, as summarized in Figure 13. The chemical exfoliation was shown to be thermodynamically favorable when Al is used as an A element, while it is unfavorable in the case of Si, with a total of 146 HE-MXenes estimated to be synthesizable. However, no competing processes during the etching were considered, which would be needed to judge whether all of the M elements are expected to endure the etching. Nevertheless, based on the computational predictions, two novel HE-MXenes were synthesized.^73^

### 3.4 Predicting MXenes with Machine Learning

For the efficient prediction of new materials, it is necessary to have descriptors that are easy to calculate at the same time as they can be correlated to synthesizability. In the previous sections the focus has been on theoretical studies attempting to explain MXene synthesis based on different ways of expressing formation energies, exfoliation energies, as well as vacancy formation energies. Also, other properties have been used in the literature, such as bond strength evaluated in different ways. The choice of properties to compare is based on reasoning and the cumulative scientific understanding up to this point and, to some extent, chemical intuition. An alternative of using the ability of the human brain is to use machine learning to elucidate patterns in data and develop criteria for synthesizability. Here, some of the work utilizing machine learning protocols to find new MXenes will be highlighted. However, we will not put emphasis on the actual machine learning algorithms but rather focus on how they have been employed.

The *positive and unlabeled* (PU) machine learning framework was used to predict which theoretically proposed MXenes have the highest probability of being synthesized.^155^ The method has its name from the fact that it relies on *positive* data, which in this case is experimentally synthesized materials, while all materials that have not yet been synthesized are treated as *unlabeled*. In other words, the algorithm has no knowledge of unsuccessful experimental attempts of, for example, the etching of a particular MAX phase. The study focused on finding both new MAX phases and MXenes, limiting the search to single-M materials. In total, 792 potential MAX and 66 MXenes candidates were considered. Notice that for each MXene candidate, 12 MAX precursors were considered, differing only by the choice of A element. The machine learning algorithm was fed with various calculated properties, and the algorithm decided which are the most important ones (each property gets a rank from the machine learning). For MXene synthesis, the five features the algorithm deemed of highest importance for synthesizability were (1) M–X bond distance, (2) cohesive energy, (3) X atom Bader charge, (4) formation energy, and (5) mass per atom. The algorithm gave a synthesizability criterion of both MAX phase and resulting MXene. In addition, a third criterion was added by hand, based on mechanical exfoliation energy. Combining these three criteria, a list of 20 MAX/MXene combinations were considered to have the highest synthesizability, with the most probable MXene being Zr_2_C synthesized from Zr_2_GaC. As highlighted in this Review, the mechanical exfoliation energy is not a valid descriptor to estimate exfoliability, making the conclusions disputable. One could also question the human intervention by including the mechanical exfoliation energy as a criterion by hand instead of asking the machine learning algorithm to rank its relevance for predicting synthesizability. Nevertheless, with the recent advances made to theoretically understand the synthesis of MXenes, it would be interesting how other calculated properties, such as chemical exfoliation energies and vacancy formation energies, would affect the outcome of such a machine learning algorithm.

Other machine learning approaches for MXenes have not focused on predicting new synthesizable MXenes but rather on how to efficiently associate the structure of a MXene with various properties. For example, Li and Barnard used a multitarget machine learning algorithm to predict the relationship between the composition of a MXene and electrochemical properties.^177^ In addition, it was shown how the process could be reversed, to predict the formula for MXenes based on preselected battery performance criteria. Machine learning has also been used to make accurate band gap predictions of functionalized MXenes.^156^

In this Review, the focus is on the synthesis of MXenes rather than their properties. Although we will not go into more detail about how machine learning can accelerate the prediction of MXene learning, it is clear that it has the potential to aid the discovery of novel materials with desired properties, in particular if it were to be combined with a robust theoretical approach to predict synthesizability.

## 4 MXene Preparation

**Figure 14:**
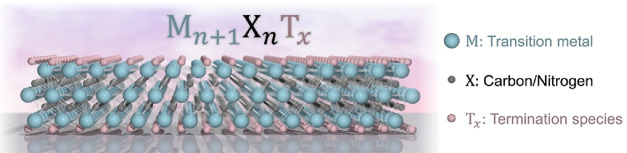
Schematic of a typical single sheet of a MXene. The MXene has the general formula M_*n*+1_X_*n*_T_*x*_, where M is an early transition metal, X is carbon and/or nitrogen, and T_*x*_ represents surface terminations that are present on both outer sides of the MXene sheet.

When a MXene is synthesized, the surface is covered by surface terminations, O, OH, F, Cl, Br, S, Se, Te, and/or NH;^1,7,178^ see Figure 14. These can be of a mixed character or in the form of single species, depending on the MXene composition and the choice of synthesis procedure. The terminations can be removed or changed through post-etching procedures,^7,179^ which in turn can have a drastic effect on the materials properties. Since the present Review focuses on the design and synthesis of MXenes, we will direct our attention toward MXenes with a structure and composition inherent to specific synthesis methods, and we will not discuss post-processing of these materials to change the surface chemistry.

### 4.1 Choice of Synthesis Method

**Figure 15:**
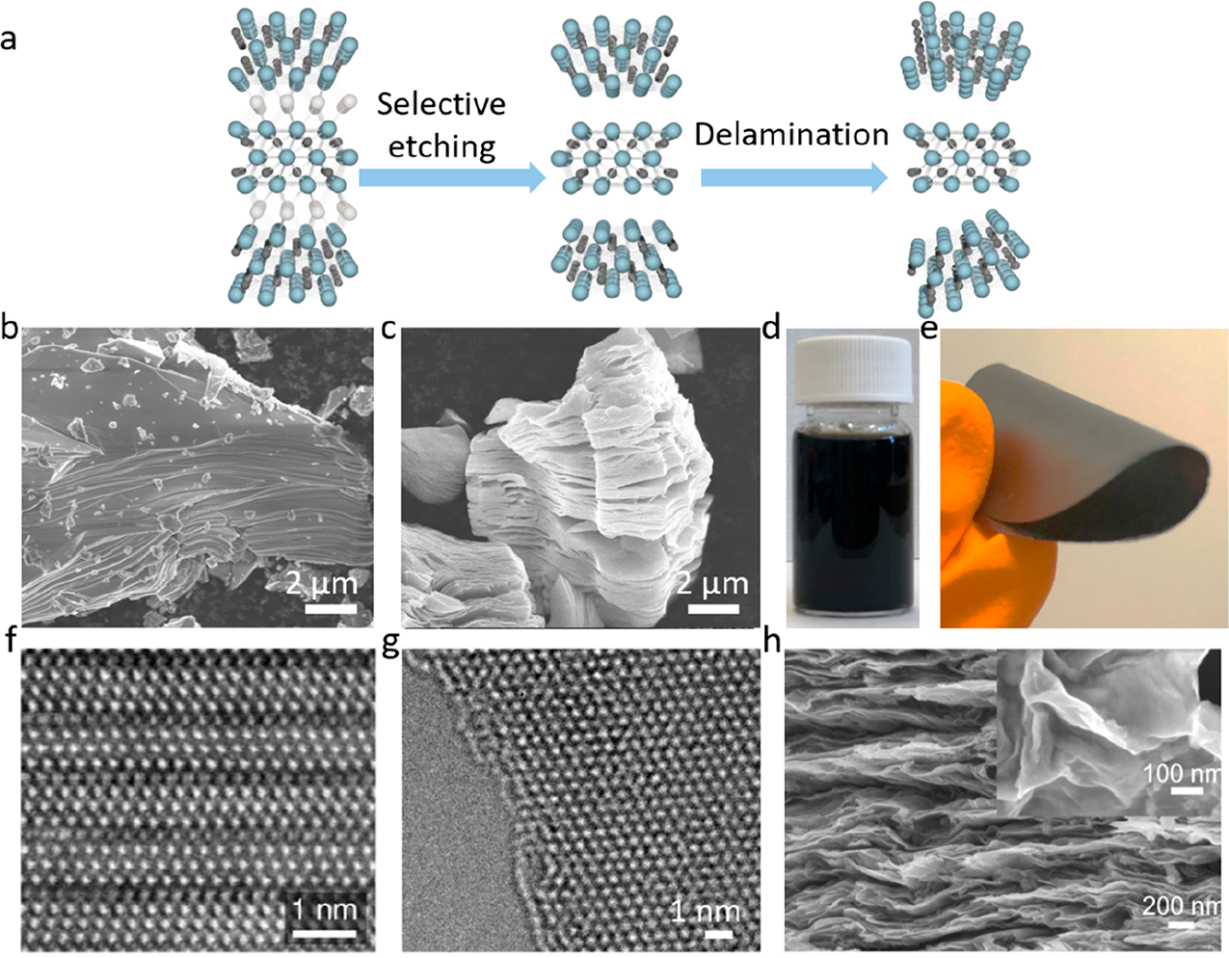
Common synthesis and processing of MXenes. (a) Schematic illustration of a wet chemical etching procedure to produce MXenes by removal of A layers from a MAX phase (terminations not shown). In this method, the MAX phase is typically selectively etched in fluorine-containing acids, resulting in multilayered MXene particles or in situ delaminated 2D flakes (using the MILD method). (b) Scanning electron microscope (SEM) image of a laminated Ti_3_AlC_2_ MAX phase crystal. (c) SEM image of a multilayer Ti_3_C_2_T_*x*_ MXene particle, derived from Ti_3_AlC_2_ by selective etching of Al layers in hydrofluoric acid (HF), showing typical accordion-like morphology. (d) Colloidal aqueous suspension of delaminated Ti_3_C_2_T_*x*_ flakes. (e) Digital photograph of a vacuum filtered flexible film of Ti_3_C_2_T_*x*_ MXene. (f) Side-view STEM image of the Ti_3_AlC_2_ MAX phase crystal. (g) Top-view atomic resolved STEM image of a single-layer Ti_3_C_2_T_*x*_ sheet. (h) Cross-sectional SEM image of a filtered film of Ti_3_C_2_T_*x*_, showing the morphology of stacked sheets. (b, c, d, e, h) Unpublished results. (f, g) Courtesy of P. O. Å. Persson.

MXenes are typically produced by top-down synthesis from its MAX phase precursors.^1,4^ As discussed in section 2, MAX phases are laminated solids with interlayer interactions much stronger than van der Waals forces, which does not allow direct mechanical exfoliation of single M_*n*+1_X_*n*_ layers from the parent MAX phases. A selective etching procedure, also sometimes referred to as chemical exfoliation, is thus required, enabled by the difference in interlayer bonding and chemical reactivity of individual layers, also associated with soluble A layer containing products (Figure 15a). The metallic bonding between M and A layer atoms in many MAX phases is weaker than the ionic and/or covalent bonding present between M and X atoms.^2^ This bonding characteristic enables the selective breaking of M–A bonds through thermodynamically favorable chemical reactions in appropriate etchants, cleaving the 3D MAX phase into terminated 2D M_*n*+1_X_*n*_T_*x*_ layers with an accordion-like multilayer structure, where the 2D MX layers are held together by hydrogen and van der Waals bonds.^1^ After the etching is finished (that is, after complete removal of the A layers), washing is required to remove residual acid and reaction products (salts) and to achieve a safe pH (∼6).^1^ Washing is normally done by repeated centrifugation to separate multilayered MXene from acidic solution and decantation of the acidic supernatant. After the pH is increased to ∼6, the multilayered flakes can be collected by vacuum-assisted filtration and vacuum drying. To further obtain single/few-layer MXenes, intercalation and delamination steps are performed.

In 2011, the first MXene, multilayer Ti_3_C_2_T_*x*_, was discovered through selective etching of Al from Ti_3_AlC_2_ MAX phase in concentrated aqueous hydrofluoric acid.^1^ Since then, the rise of MXenes has given birth to nearly 50 stoichiometric MXene compositions (Table 1). The design and synthesis of *o*-MAX, *i*-MAX, and multi-M element MAX phases, as well as MAX phase related laminated solids, have created a playground for the design of corresponding new MXene materials, hence further expanding the growing family of MXenes. In this section, we focus on the methods for MXenes preparation, ranging from the main method to date, wet chemical etching, to more recent advances such as direct CVD growth. Among different developed etching techniques, the wet chemical etching of MAX phases in fluoride-containing acidic solutions results in the highest yield of the MXene materials to date.

#### 4.1.1 MXene Synthesis by Use of Aqueous Hydrofluoric Acid

**Figure 16:**
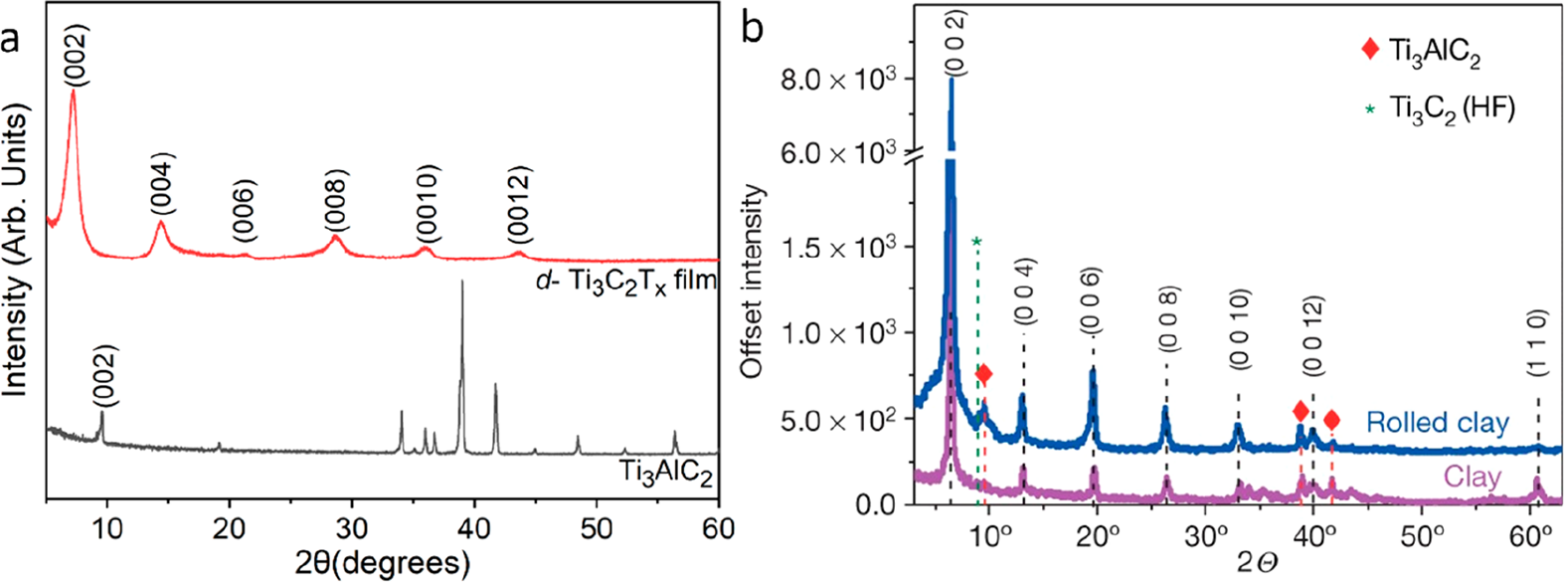
(a) XRD patterns of Ti_3_AlC_2_ powder and a film of delaminated Ti_3_C_2_T_*x*_ sheets produced by etching in an HF solution. (b) XRD patterns of samples produced by etching in LiF-HCl solution. Multilayer Ti_3_C_2_T_*x*_ produced by etching in LiF-HCl solution, referred to as clay, shows a sharper, intense (002) peak and higher order (00*l*) peaks. The (002) peak of MXene produced by this method is also at a much lower angle than that for a typical of MXene produced by HF (vertical green line). (a) Unpublished result. (b) Reproduced with permission from ref (5). Copyright 2014 Springer Nature.

Different concentrations of aqueous hydrofluoric acid (HF) are commonly used to etch Al-containing MAX phases.^1,4,35,44,55,84^ Removal of the A layer atoms in aqueous HF and the concomitant surface functionalization of the M_*n*+1_X_*n*_ layers results in lattice expansion along the *c* axis, as evident from the apparent broadening and downshift of the X-ray diffraction (XRD) peaks corresponding to (00*l*) planes of the MAX phases (Figure 16a).^96^ If etching is efficient and all of the MAX phase is gradually transformed into MXene, the corresponding XRD peaks of the MAX phase precursor lose their intensity and ultimately disappear, being replaced by broad reflections from the basal planes of the MXene. The selective etching in aqueous HF results in separation of the 2D M_*n*+1_X_*n*_ layers and their spontaneous termination with surface functional groups in the form of −O, −F, and −OH, which most likely reduce their chemical potential and increase their thermodynamic stability (eqs 8–11).

Generally, HF has proven to be an efficient etchant for the selective removal of Al from MAX phase precursors, but at the same time it is a very corrosive chemical, which imposes strict procedures for the material synthesis. The MXene produced by using HF as the etchant usually results in the formation of multilayer MXene particles with a typical accordion-like morphology (Figure 15c),^1,4^ likely due to the evolution of hydrogen gas during synthesis. The produced multilayer MXene can subsequently be delaminated into single- or few-layer flakes (Figure 15g) through further chemical intercalation of small organic molecules such as dimethyl sulfoxide (DMSO),^1^ tetrabutylammonium hydroxide (TBAOH),^180^ tetramethylammonium hydroxide (TMAOH),^181^ or *n*-butylamine.^180^ Inorganic salts, such as LiCl,^182^ can also be used as an intercalant. Because of their predominantly anionic surface terminations, delaminated MXenes produced by wet chemical etching have a zeta potential below −30 mV and can form stable colloidal solutions (Figure 15d). The solution can easily be vacuum filtered into a flexible film (Figure 15e) for further characterization or property testing. Taking the selective etching of Al from Ti_3_AlC_2_ in aqueous hydrofluoric acid as an example,^1^ the general chemical reactions can be approximated by the following simplified equations:
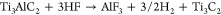
8
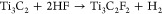
9
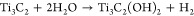
10
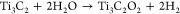
11

#### 4.1.2 In Situ HF for MXene Derivation

**Figure 17:**
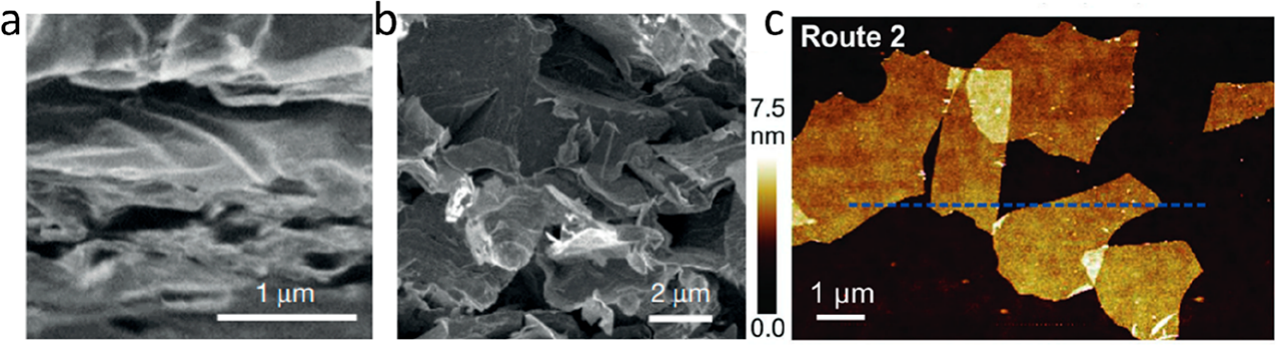
(a–b) SEM images of multilayer Ti_3_C_2_T_*x*_ MXene from etching in LiF-HCl solution.^5^ (c) AFM images of Ti_3_C_2_T_*x*_ MXene flakes produced by the MILD method, deposited on Si/SiO_2_ substrate.^184^ (a–c) Reproduced with permission from refs (5 and 186). Copyright 2014 Springer Nature and 2021 Wiley-VCH, respectively.

Etching in alkali fluoride salts (LiF, NaF, KF, etc.) mixed with hydrochloric (HCl) acid or other acids is another approach that provides a safer and milder pathway for MXene synthesis.^5,183^ In this approach, also known as the MILD method, the mixing of HCl and metal fluoride results in *in situ* formation of HF and an intercalant (such as Li ions if a LiF salt is used, see eq 12), and therefore etching and intercalation take place simultaneously. Also, because of the presence of Li cations between flakes, the MXene flakes produced by the MILD method show clay-like behavior.^5^ It should be noted that multilayer Ti_3_C_2_T_*x*_ MXene produced by this method shows a tightly stacked morphology (Figure 17a–b),^5^ in contrast to the accordion-like morphology observed in HF-produced multilayer MXenes (Figure 15c), probably as a result of water and/or cationic intercalation. The MILD method is only applicable to a few MXenes to date,^5,35,71,72,84^ for example, Ti_2_CT_*x*_, Mo_2_CT_*x*_, Mo_4/3_CT_*x*_, and W_4/3_CT_*x*_, but the flake size and quality (defect concentration and conductivity) are better controlled compared to other methods. Through fine-tuning of the molar ratio/concentration of fluoride salt and acid, Ti_3_C_2_ flakes with a lateral size of up to a few micrometers can be achieved (Figure 17c).^181^ As metal cations are intercalated simultaneous to the actual etching, a MXene produced by the MILD method can be turned into single flakes via sonication or hand-shaking,^5,183^ without the use of organic intercalants.
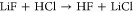
12

Wet chemical etching techniques have produced MXenes from Al-containing MAX phases and related ceramics (non-MAX phases), with a few exceptions, where Ga double-layers,^34^ Al_3_C_3_,^39^ [Al(Si)]_4_C_4_ carbide layers,^38^ or Si atomic layers^31^ were removed using a mixture of HF and oxidants.

#### 4.1.3 Bifluoride, Organic Base, and Halogen Etchants

The wet chemical etching method has been further extended to utilize also NH_4_HF_2_,^185^ aqueous tetramethylammonium hydroxide (TMAOH) solution,^181^ and halogen etchants.^186,187^ In addition to the use of an aqueous solution, efforts have also been made to produce MXene in a nonaqueous solution. Ammonium dihydrogen fluoride (NH_4_HF_2_) dissolved in polar organic solvents (e.g., propylene carbonate) has been applied for the synthesis of highly fluorinated Ti_3_C_2_T_*x*_ MXene,^188^ and a larger interlayer spacing of 21–51 Å (depending on the solvent) was obtained due to the cointercalation of NH_4_^+^ cation complexes with the solvent molecules. Generally, nonaqueous etchants enable the synthesis of highly fluorinated MXene, compared to O-rich terminations obtained when water is the etching medium, which also pave the way for using MXenes in numerous water-sensitive applications.

Recently, Ti_3_C_2_Br_2_ has been synthesized by etching of Ti_3_AlC_2_ in etchants based on halogen compounds, i.e., tetrabutylammonium bromide in anhydrous cyclohexane.^187^ Fluorine-free and oxygen-free Ti_3_C_2_T_*x*_ was also obtained by using iodine dissolved in anhydrous acetonitrile (CH_3_CN).^186^ Reducing or eliminating the use of fluoride-containing etchants is expected to produce MXene with less defects and more controllable terminations.

#### 4.1.4 MXene Derivation Based on Molten Salt

In addition to the above-mentioned wet chemical techniques, efforts have recently been made to prepare MXenes in molten salt media, through redox potential coupling of A layers with the late transition-metal halides (eg., CuCl_2_, CdBr_2_, and CuI),^7,189^ which are so-called Lewis acids in their molten states. The A layer atoms with lower redox potentials can be etched out from the MAX phase precursor by the cations in the molten salt, being of a higher redox potential, obtaining −Cl, −Br, and −I terminated MXenes after an inevitable washing step. MAX phases with a variety of A layer elements (Al, Zn, Si, Ga, etc.) can be transformed through this process into MXenes at 550–750 °C. In such F-free MXenes, the bond strength of the M–Cl or M–Br bonds is weaker than those of M–F or M–O bonds, which paves the way for the controllable modification or removal. For example, the halogen terminations can be further tailored toward −S, −Se, and −Te surface terminations through post-substitution reactions in a eutectic melt system,^7^ resulting in changed electronic properties, including superconductivity. Taking Ti_3_SiC_2_ as an example, the weakly bonded A layer atoms (Si) can be easily converted to Si^4+^ by a redox reaction (eq 13) in the acidic molten CuCl_2_ environment, resulting in the formation of the volatile SiCl_4_ (T_boiling_ = 57.6 °C) gas phase and the reduction of Cu^2+^ to Cu metal (eqs 13–14).^189^ The exposed Ti atoms are further saturated by Cl^–^ anions to form Ti_3_C_2_Cl_2_.
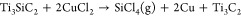
13
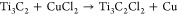
14

**Figure 18:**
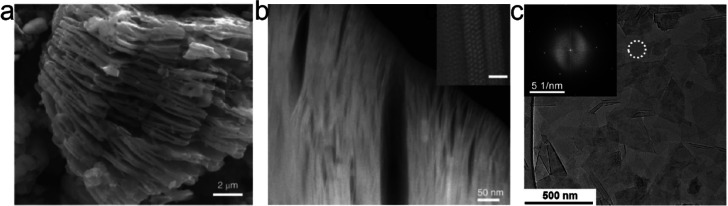
(a) SEM image of a multilayer Ti_3_C_2_Cl_2_ MXene produced by selective etching of Ti_3_SiC_2_ in molten CuCl_2_. (b) Cross-sectional STEM image of the multilayer Ti_3_C_2_Cl_2_ MXene. The inset in (b) is an atomically resolved image with a scale bar of 1 nm.^189^ (c) TEM image of Ti_3_C_2_Cl_2_ MXene flakes (produced by selective etching of Ti_3_AlC_2_ in molten CdCl_2_) deposited from a colloidal solution. The inset in (c) is a fast Fourier transform of the circled region, showing crystallinity and a hexagonal symmetry of the individual Ti_3_C_2_Cl_2_ MXene flake. (a–c) Reproduced with permission from refs (7 and 189). Copyright 2020 American Association for the Advancement of Science and 2020 Springer Nature, respectively.

As the interaction between the halogen terminations (−Cl in this case) is stronger than the interaction between O and/or OH, the ordering along the *c*-axis in the as-produced multilayer Ti_3_C_2_Cl_2_ is much higher than in MXenes produced in fluoride-containing solutions, evidenced by the very sharp and intense (00*l*) XRD peaks.^189^ Water is typically used to dissolve the residual salts, and additional washing steps in suitable chemicals are needed to remove byproducts like reduced metals or partially reduced salts, and the nature of these chemicals may influence the surface of the molten salt derived MXenes. Due to the stronger interaction between the multilayer halogen-terminated MXenes derived from molten salt, it is still challenging to produce free-standing halogen-terminated MXene sheets at a larger scale (Figure 18a–b). Still, the first delamination of Ti_3_C_2_Cl_2_ (derived from etching of Ti_3_AlC_2_ in molten CdCl_2_) was achieved by intercalation of *n*-butyl lithium (*n*-BuLi), with subsequent dispersion in a polar organic solvent in the form of *N*-methylformamide (NMF) (Figure 18c).^7^ Furthermore, in a recent work, delamination of Ti_3_C_2_Cl_2_ (derived from etching of Ti_3_AlC_2_ in molten CuCl_2_) was performed via intercalation of the organic molecule TBAOH, followed by sonication to separate the layers.^190^

#### 4.1.5 Organic Base As an Etchant and the Surface Acoustic Wave Method

In addition to acids or acidic molten salts, an organic base such as tetramethylammonium hydroxide (TMAOH) has also been employed for the preparation of Ti_3_C_2_T_*x*_ MXene,^181^ taking advantage of the amphoteric nature of the interlayer Al (eq 15). The organic base acts as both an etchant and an intercalant. The resulting aluminum-oxoanion-functionalized titanium carbide sheets exhibit strong optical absorption in the near-infrared (NIR) region. For further advancement of synthesis approaches, rapid synthesis of Ti_3_C_2_T_*x*_ (in milliseconds) by exploiting the nonlinear electromechanical coupling afforded by surface acoustic waves (SAWs) is a recent effort toward cost-effective preparation of MXenes.^191^ Inducing self-ionization of pure water to facilitate the production of free radicals (protons) in the absence of any catalysts, the protons combine with fluorine ions from LiF to produce *in situ* HF. This, in turn, selectively etches the MAX phase into MXene, whose delamination is aided by the strong acoustic forcing.
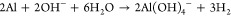
15

#### 4.1.6 Synthesis in Hydrogen Chloride Gas Media

Very recently, efforts have been made to prepare MXenes by a reaction of MAX phases in hydrogen chloride (HCl) gas media.^192^ For example, Ti_4_N_3_Cl_2_ MXene has been prepared by the reaction between Ti_4_AlN_3_ MAX phase and HCl gas (eq 16). In contrast to the previously discussed approaches in liquid or molten medium, reactions in the gas phase enable faster speed through thermodynamic driving forces for removal of the A layers. This is due to the negative Gibbs free energies for reactions between HCl gas molecules and A layer atoms over a wide temperature range, from 800 to 1500 K, and the high vapor pressure of the formed gaseous byproducts ACl_*x*_ (AlCl_3_ when etching Ti_4_AlN_3_ in HCl gas media). Moreover, this process is easy to scale up to a level on the order of 10 kg, which is promising for future large-scale manufacturing. The resulting multilayer Ti_4_N_3_Cl_2_ MXene exhibits a loosely packed multilayer structure, probably originating from the rapid removal of the gaseous byproduct AlCl_3_ during the etching process, which can be exfoliated into 2D sheets via a sonication treatment.
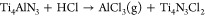
16

#### 4.1.7 Direct CVD Growth of MXenes

**Figure 19:**
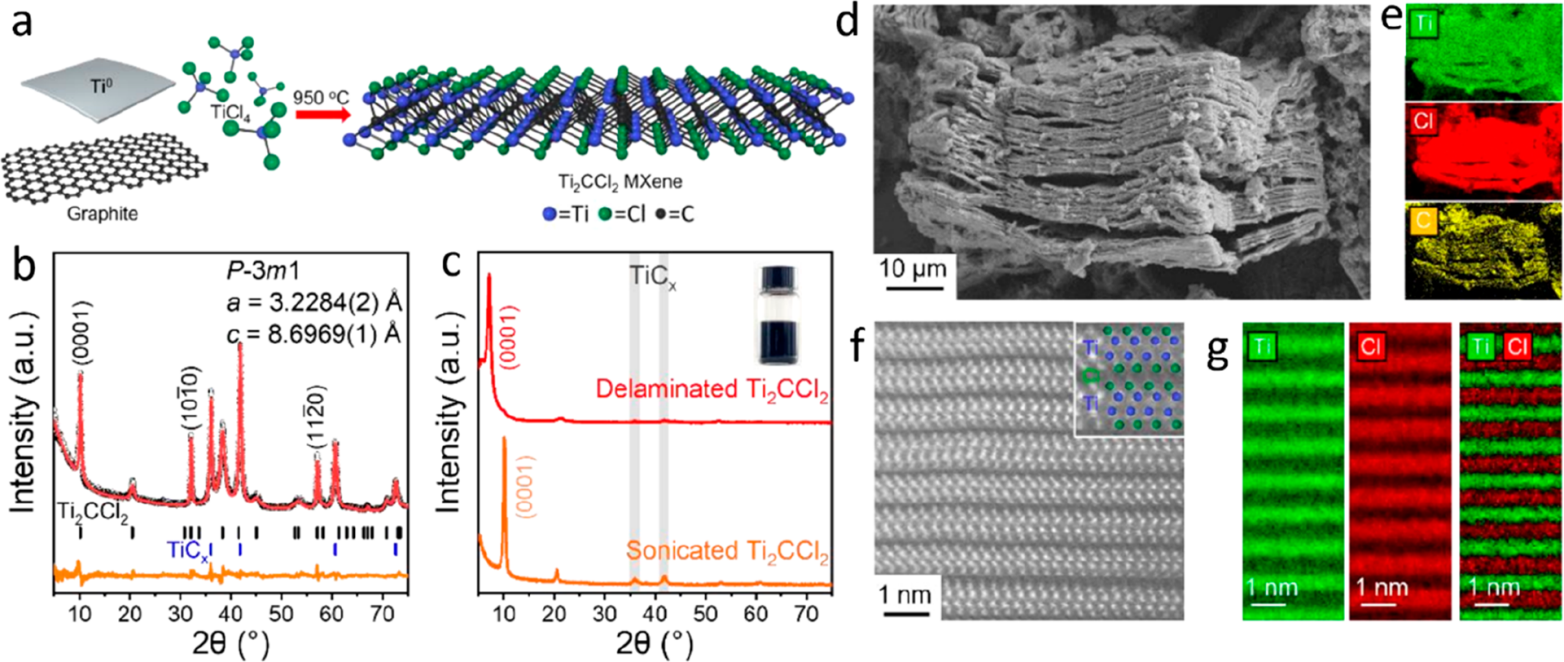
Direct synthesis (DS) and characterization of Ti_2_CCl_2_ MXene (here denoted DS-Ti_2_CCl_2_). (a) Schematic diagram of the synthesis. (b) XRD pattern and Rietveld refinement of DS-Ti_2_CCl_2_ prepared by reacting Ti, graphite, and TiCl_4_ at 950 °C. (c) XRD patterns of dispersible delaminated and sonicated DS- Ti_2_CCl_2_ MXenes. Inset: Colloidal solution of the delaminated DS-Ti_2_CCl_2_. (d) SEM image and (e) EDX elemental mapping of a DS-Ti_2_CCl_2_ stack. (f) High-resolution HAADF image and (g) EELS atomic column mapping representing the layered structure of DS-Ti_2_CCl_2_. (a–g) Reproduced with permission from ref (193). Copyright 2023 American Association for the Advancement of Science.

In addition to the development in the past decade of the general top-down synthesis of MXenes from their MAX phase or MAX-phase-like precursor, the most recent efforts have been directed toward direct synthesis of MXenes (bottom-up synthesis) by reactions of metals and metal halides with graphite, methane, or nitrogen (eq 17 and Figure 19a–g).^193^ The development of direct synthetic methods is expected to facilitate a broadening of future areas for MXene applications. The direct chemical vapor deposition (CVD) growth route enables growth of MXenes with unique morphologies and MXenes that have not been synthesized from MAX phases. For example, Ti_2_CCl_2_ MXene was prepared by CVD at 950 °C on a Ti surface with a CH_4_ and TiCl_4_ gas mixture diluted in Ar with 15 min exposure.^193^ The directly synthesized MXenes can be delaminated through intercalation of *n*-butyl lithium (*n*-BuLi) (Figure 19c), with subsequent dispersion in a polar organic solvent.^193^
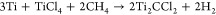
17

## 5 MXenes Synthesized to Date

### 5.1 Inherent Structure and Composition

**Figure 20:**
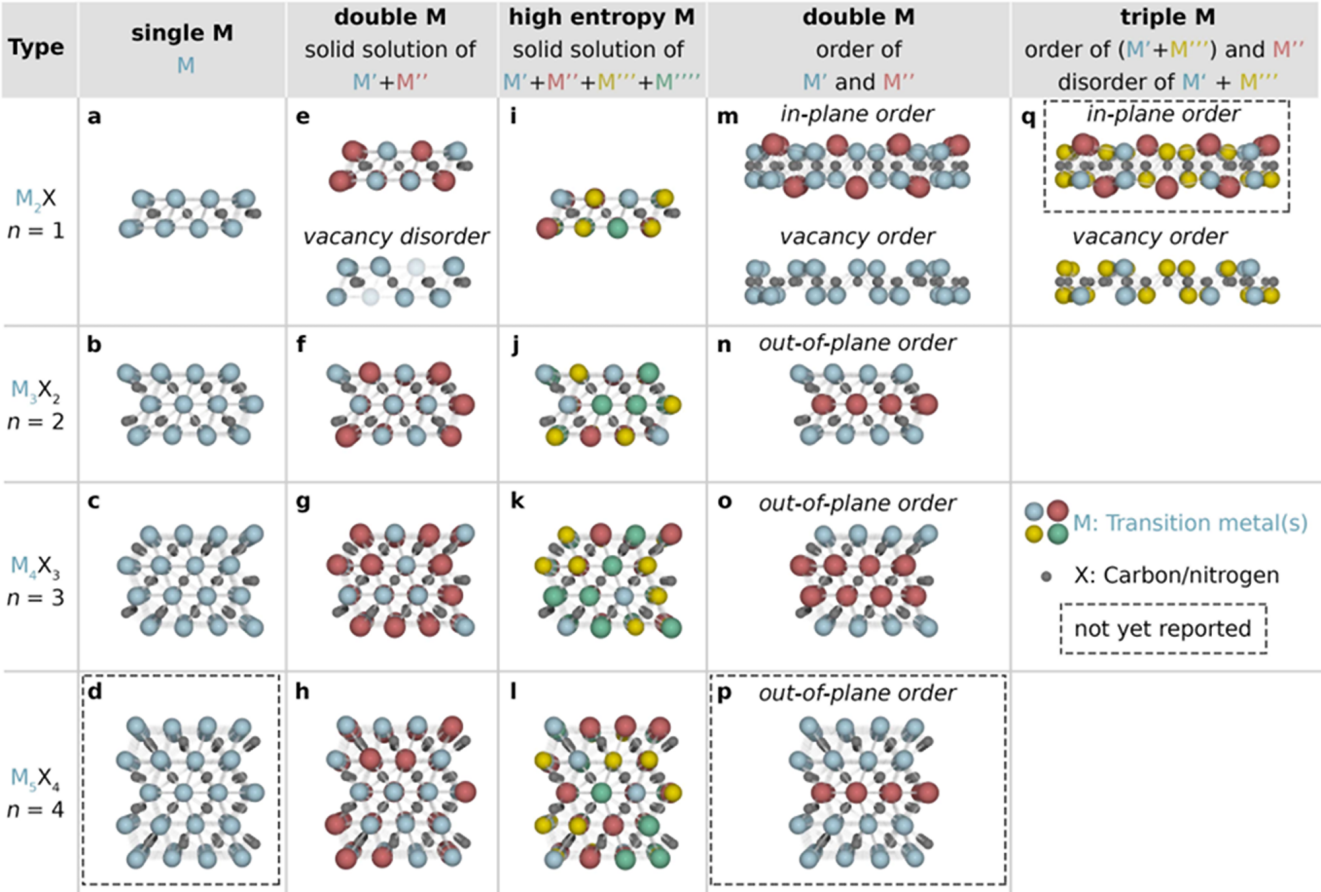
Schematic illustration of the MXenes structures (excluding surface terminations). MXenes have a general formula of M_*n*+1_X_*n*_T_*x*_, where M is an early transition metal, X is carbon and/or nitrogen, and T_*x*_ denotes surface groups, such as −O, −F, −OH, −Cl, −Br, −Se, −S, and −Te. The value of *n* ranges from 1 to 4, depending on the number of transition metal carbide and/or nitride layers M_*n*+1_X_*n*_ present in the MXene structure. The metal (M) sites of MXenes can be occupied with (a–d) one, (e–h, m–p) two, or (i–l, q) multiple transition metal atoms, being arranged (e–l) randomly, forming a solid solution disorder, or (m–q) occupying specific sites, giving rise to chemically out-of-plane or in-plane ordered MXene structures.

The rise of MXenes during the past decade has given birth to more than 50 different MXene stoichiometries; see summary in Table 1 along with corresponding precursor materials. Figure 20 provides a schematic overview of experimentally realized MXenes to date. Most of these materials are derived from bulk MAX phases that are structurally defined by M_*n*+1_X_*n*_ layers interleaved by one layer of group 11–16 A element atoms. The composition of the MXene is thus highly dependent on the MAX phase precursor. Traditional MXenes are, as mentioned before, described by the formula M_*n*+1_X_*n*_T_*x*_; see Figure 20 a–d, where M is an early transition metal, X is C and/or N, and T_*x*_ denotes surface terminations, such as −O, −F, −OH, −Cl, −Br, −Se, −S, and −Te. The atoms on the M site can be composed of single, double, triple, or multiple (high-entropy) elements. The most studied MXene, Ti_3_C_2_T_*x*_ was discovered in 2011, through selective etching of Al from the Ti_3_AlC_2_ precursor in concentrated HF solution. Since then, 17 mono-M MXenes have been reported, based on the metals Ti, V, Nb, Mo, Ta, W, Zr, and Hf (Table 1).

#### 5.1.1 MXene Alloys

Alloying has been a prevalent concept for incorporating advantageous metals and for improving the material properties. Metal alloying of original MAX phases has also been well studied, enlarging the attainable compositions and properties of corresponding MXenes. When MAX phases with double transition metals on the M site are used as precursors, various kinds of MXenes can be obtained. As discussed in section 2, alloying on the M site in a MAX phase typically results in chemically disordered solid solutions, and therefore MXenes with a double-metal solid solution are formed, denoted as (M′,M′′)_*n*+1_X_*n*_T_*x*_, if both M elements are preserved during the etching process. As listed in Table 1, 15 types of (M′,M′′)_*n*+1_X_*n*_T_*x*_ have been reported to date, encompassing *n* from 1 (e.g., (V_1–*x*_Nb_*x*_)_2_C)T_*x*_ to 4 (Mo_1–*x*_V_*x*_)_5_C_4_)T_*x*_, with half of them being (M′_1–*x*_M′′_*x*_)_4_C_3_T_*x*._ Control of the composition of the double-M MAX phase will impact the composition and properties of the double-metal solid solution MXenes. For example, Han et al. clearly demonstrated this when tuning the M′/M′′ ratio in the MAX phase, which allows for control of electronic and optical properties in the corresponding (Ti_1–*x*_V_*x*_)_2_CT_*x*_ and (Ti_1–*x*_Nb_*x*_)_2_CT_*x*_ solid solution MXenes.^60^

The ratio of M′ and M′′ in a solid solution MAX phase is in general unchanged in the corresponding double-metal MXene. However, it has been shown that it is possible to selectively etch both Al and Sc from solid solution (Nb_0.67_Sc_0.33_)_2_AlC to produce Nb_1.33_CT_*x*_ MXene with disordered metal vacancies.^82^ Another example is V_2–*x*_CT_*x*_ MXenes with disordered vacancies,^83^ obtained from a (V_1–*x*_Sc_*x*_)_2_AlC (*x* ≤ 0.05) MAX phase; see schematic in Figure 20e. If the alloying element in the solid solution is removed together with Al upon etching, the formed vacancies can also cluster and form pores in the 2D sheets, similar to the pores obtained from what is sometimes referred to as overetching the MXene material.^194^ The latter process is, however, not as easily controlled.

Selective etching of MAX phases with alloying on the X site is more scarce and results in MXenes with a disordered solid solution on the X site, commonly denoted as M_*n*+1_(X′,X′′)_*n*_T_*x*_. The alloying element in these materials is currently limited to C or N, e.g., Ti_2_C_0.5_N_0.5_T_*x*_.^6^

#### 5.1.2 *o*-MXenes

Besides solid solutions, it has more recently been demonstrated that alloying on the M site of the MAX phase can also result in chemically ordered structures, including both out-of-plane chemical order (*o*-MAX) and in-plane chemical order (*i*-MAX); see section 2. The discovery of these nonconventional MAX phase alloys enables the formation of corresponding MXenes with unique structures and properties.

**Figure 21:**
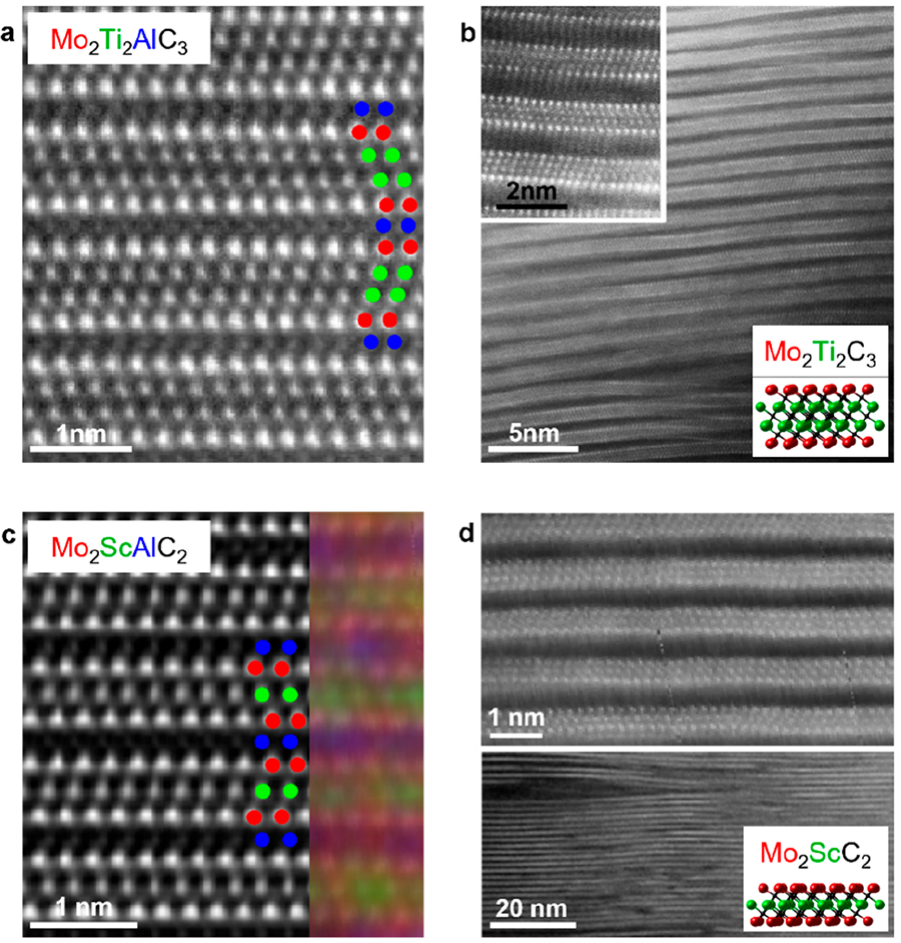
(a) HRSTEM image showing the out-of-plane ordered structure of the Mo_2_Ti_2_AlC_3_*o*-MAX phase along the [11–20] zone axis. (b) HRSTEM image of the corresponding Mo_2_Ti_2_C_3_T_*x*_*o*-MXene, where the atomically resolved image of the same MXene in the inset clearly shows the chemical order. (c) HRSTEM image showing the out-of-plane ordered structure of the Mo_2_ScAlC_2_ MAX phase along the [11–20] zone axis. (d) HRSTEM image displaying the atomic resolved Mo_2_ScT_*x*_*o*-MXene, derived from Mo_2_ScAlC_2_. (a–d) Reproduced with permission from refs (93 and 96). Copyright 2017 Elsevier and 2015 American Chemical Society, respectively.

MXenes with out-of-plane chemical order, *o*-MXenes, are obtained through selective etching of *o*-MAX phases. They are characterized by alternating layers of M′ and M′′, and the M′ and M′′ occupy specific sites resulting in out-of-plane chemical order, as shown in Figure 20n–p and Figure 21a–d. To date, these MXenes include both M′_2_M′′X_2_T_*x*_ and M′_2_M′′_2_X_3_T_*x*_ structures, as well as the compositions of Mo_2_Sc, Mo_2_Ti, Cr_2_Ti, Mo_2_Ti_2_, and Mo_2_Nb_2_.

#### 5.1.3 *i*-MXenes

**Figure 22:**
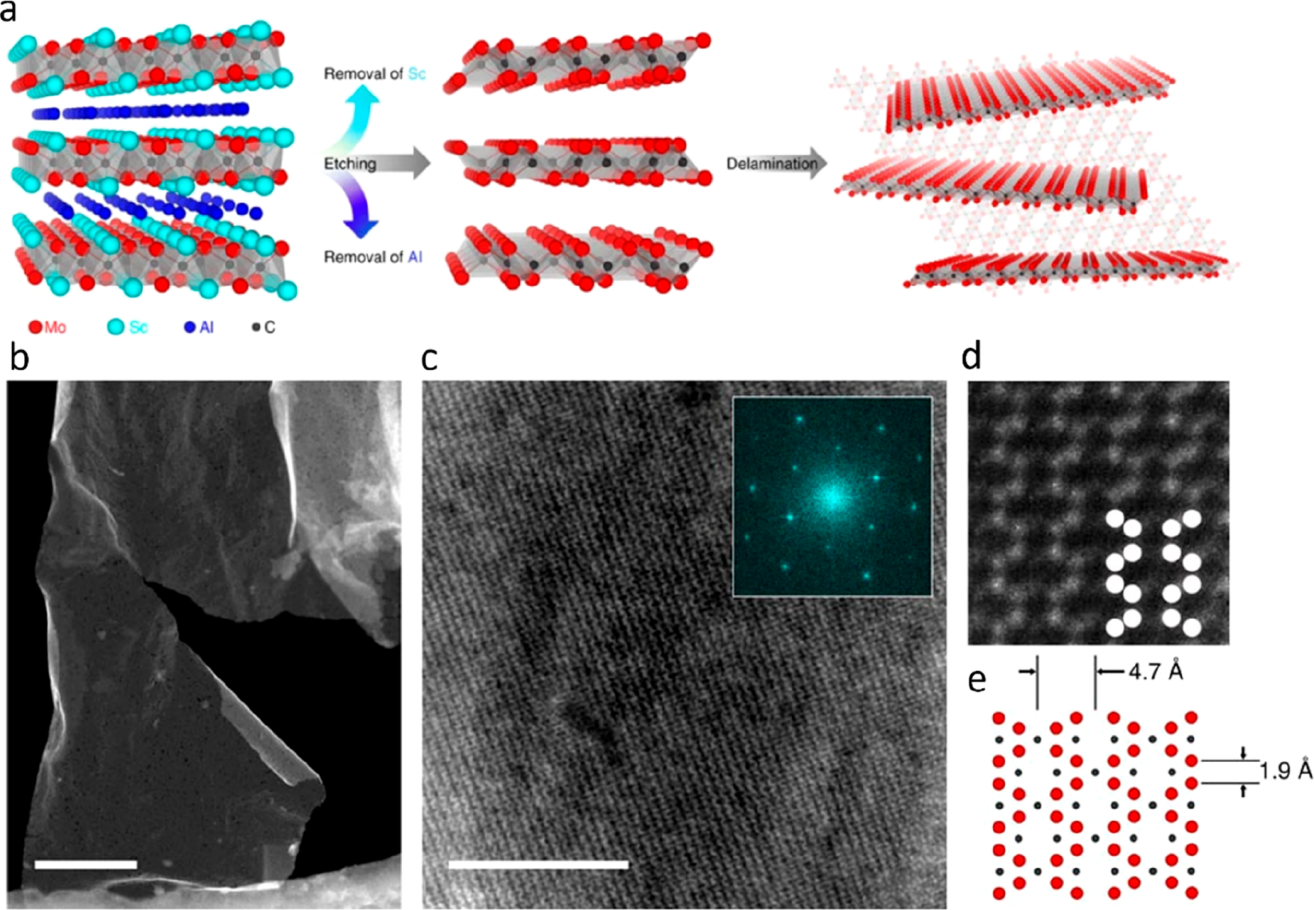
(a) Schematic of (Mo_2/3_Sc_1/3_)_2_AlC *i*-MAX before etching (left panel), after etching (middle panel), and after delamination (right panel). The background in the right panel shows the plan view of the Mo_4/3_CT_*x*_ MXene with ordered vacancies. (b) Low-magnification image of a single flake of Mo_4/3_CT_*x*_ MXene with lateral dimensions >1 μm. (c) Higher magnification, with the FFT of the original image in (b) shown in the inset. (d) Atomically resolved image with an overlaid schematic atomic structure, in comparison to (e) the ideal atomic structure derived from the theoretically simulated parent *i*-MAX phase. The scales in (d) and (e) are identical. Scale bar in (b) corresponds to 200 nm, and scale bar in (c) corresponds to 10 nm. (a–e) Reproduced with permission from ref (84). Copyright 2017 Springer Nature.

As opposed to the *o*-MXenes with out-of-plane order resembling a sandwich structure, there are MXenes with in-plane order; see Figure 20m, q. These are commonly referred to as *i*-MXenes and have the general formulas M_3/4_XT_*x*_ (a MXene with in-plane ordered vacancies),^84,88^ M′_4/3_M′′_2/3_X (a MXene with in-plane chemical order)^90^ or M′_*x*_M′′_*y*_XT_*x*_ (a MXene with in-plane ordered vacancies combined with a disordered solid solution of remaining M′ and M′′).^92^ The *i*-MXenes are obtained by chemical exfoliation of *i*-MAX phases (M′_4/3_M′′_2/3_AlC). They are thus characterized by the in-plane chemical ordering of the two M elements (M′ and M′′) in a specified 2:1 molar ratio. In *i*-MAX phases the minority element extends out from the M layer, toward the A layer, which facilitates selective removal of not only the A element but also the minority metal upon etching. The simultaneous removal of Al and M′′ upon etching (M′′ is typically Sc, Y, or a rare earth element (RE)) results in 2D *i*-MXenes with unique in-plane ordered vacancies, such as Mo_4/3_CT_*x*_ (Figure 22a–e) and W_4/3_CT_*x*_.^84,88^ However, upon carefully controlled etching, also referred to as targeted etching, it is possible to keep M′′ and maintain the in-plane chemical ordering of M′ and M′′, e.g., Mo_4/3_Y_2/3_CT_*x*_.^90^ Recently, solid solution *i*-MAX phases were demonstrated, (W_0.5_Mo_0.5_)_4/3_RE_2/3_AlC (RE = Gd, Tb, Dy, Ho, Er, and Y), composed of a solid solution disorder of W and Mo on the M′ site and RE as M′′, which upon selective etching of both Al and RE resulted in solid solution *i*-MXenes with ordered metal vacancies, (W_0.5_Mo_0.5_)_4/3_CT_*x*_.^92^

#### 5.1.4 Multielement MXenes

Extending beyond double transition metal MXenes with chemical order provides further prospects of tuning the chemical composition and therefore the properties; see Table 1. One such example is MXenes with triple transition metals (e.g., Ti_2_V_0.9_Cr_0.1_C_2_T_*x*_), where it has been found that the relative content of Cr in such a MXene has a crucial influence on the electrochemical performance.^75^ Furthermore, inspired by the concept of high-entropy alloys (HEAs), originally developed for selected oxides and carbides, the designs of the HE MAX phases and the corresponding HE MXenes have also been explored, as schematically shown in Figure 20 i–l. MXenes with four or more transition metals are generally classified as HE MXenes, and 7 HE MXenes have been reported to date.^71,73,76,77^ It has been found that the mechanical strain within the (Ti_1/5_V_1/5_Zr_1/5_Nb_1/5_Ta_1/5_)_2_CT_*x*_ MXene can promote uniform growth of lithium without dendrite formation.^71^ Furthermore, (Ti_1.1_V_0.7_Cr_*x*_Nb_1.0_Ta_0.6_)_4_C_3_T_*x*_ HE MXene based electrodes show high potential as electrode materials in supercapacitors (490 F/g at 2 mV/s).^77^ Moreover, it has been demonstrated that the HE MXene order can be tailored by the Cr content during synthesis,^77^ targeting M_2_C-type or M_4_C_3_-type, which may open additional pathways for the discovery of new materials and properties.

**Figure 23:**
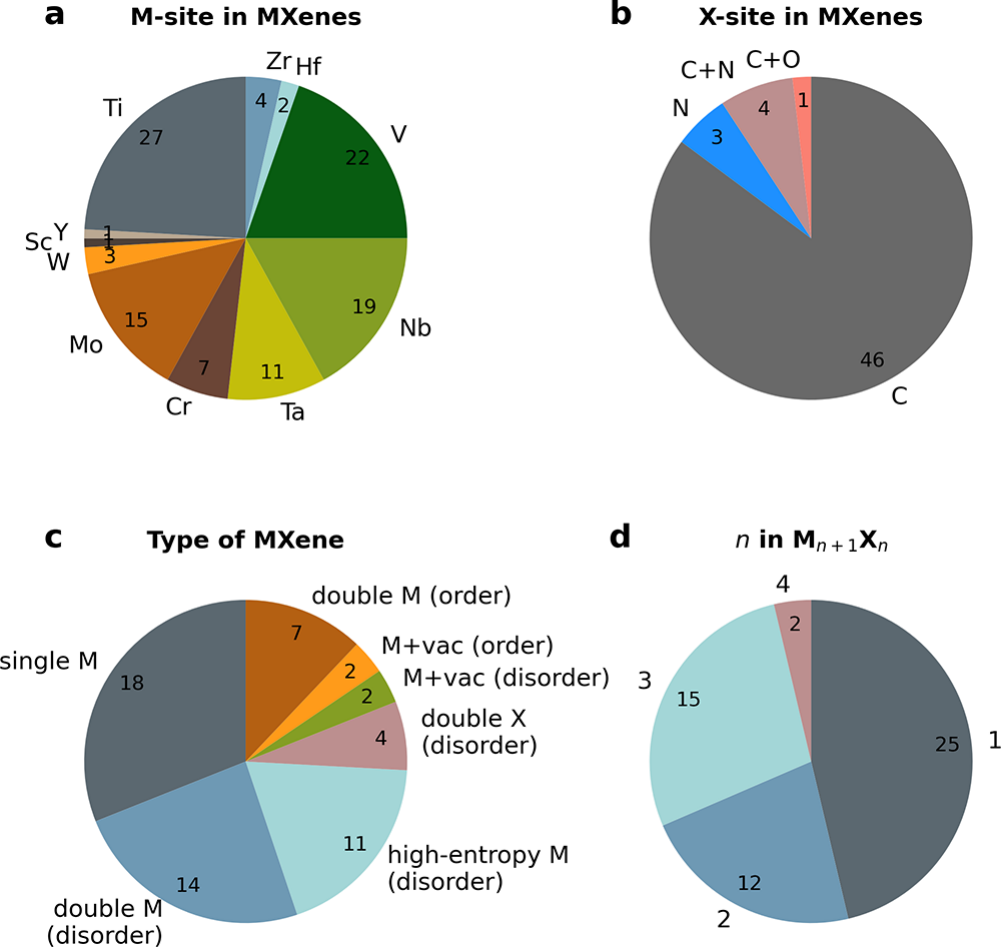
Overview of synthesized MXenes to date, based on occurrence of elements on (a) M and (b) X sites. (c) Distribution of elements (vacancies) on the M or X site. (d) Overview of MXene layer thickness for the compositions synthesized to date, given through the value of *n* in the formula M_*n*+1_X_*n*_T_*x*_. Data are based on Table 1.

Summarizing the compositions of MXenes synthesized to date, they span over 11 different transition metals (Figure 23a)—from Sc and Y in group 3 to Cr, Mo, and W in group 6—and can be composed of one (single M), two (double M), or more metals (high entropy), metal(s) combined with metal vacancies, as well as one (single X) or two (double X) elements on the X site (Figure 23b). Moreover, MXenes with double M can have the metals randomly distributed in a solid solution across all M layers or be of an ordered character (Figure 23c). In addition, considering the number of metal layers in different MXenes, given by *n*+1 in the formula M_*n*+1_X_*n*_ (*n* = 1–4), it is evident that most of the MXenes have two metal layers (*n* = 1), followed by four (*n* = 3) and three (*n* = 2) (Figure 23d).

### 5.2 MXene Morphology

**Figure 24:**
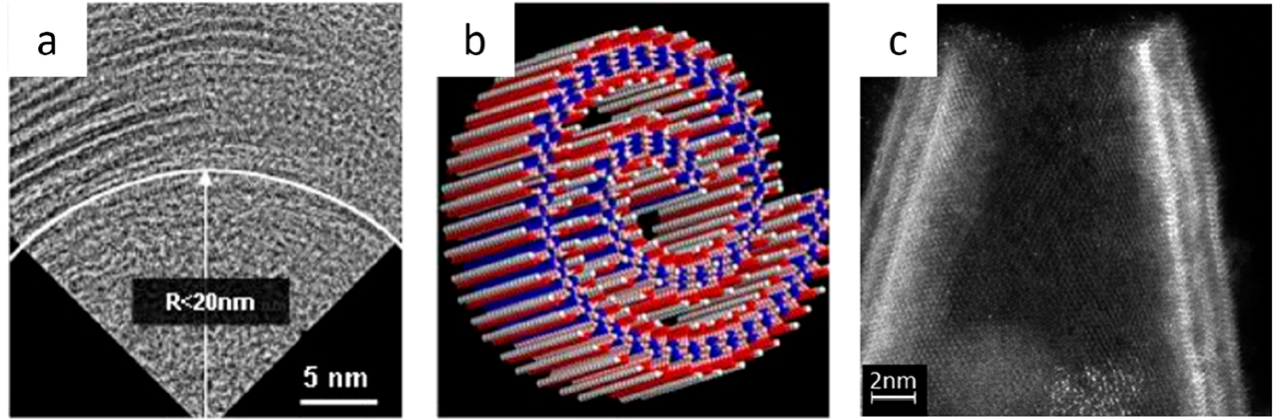
(a) Cross-sectional TEM image of a Ti_3_C_2_T_*x*_ scroll with an inner radius of less than 20 nm.^1^ (b) Corresponding schematic for a MXene scroll (with −OH termination, Ti_3_C_2_(OH)_2_).^1^ (c) HRSTEM showing a cross section of a single scroll with three V_2–*x*_CT_*x*_ MXene layers. (a–c) Reproduced with permission from refs (1 and 83). Copyright 2011 Wiley-VCH and 2019 Royal Society of Chemistry, respectively.

Generally, the morphology of as-prepared MXenes is highly dependent on the preparation method and the chosen etchants, but it is important for further processing into forms suitable for potential devices or for attainable properties required for a specific application. Loosely packed or tightly stacked multilayer particles are obtained through wet chemical etching or molten salt methods, as shown in section 5.1. Freestanding, single-layer MXene sheets or scrolls (Figure 24 a–c) can be obtained through further exfoliation, by intercalation and delamination, or by mild hand-shaking.^180,183^ MXenes can be processed into various shapes and morphologies, as shown for Ti_3_C_2_T_*x*_ in Figure 15d–e in section 4. The colloidal suspension of MXenes in water can be processed into fibers or freestanding films using vacuum-assisted filtration and can be printed into patterns without any additives. Spin-coating and dip-coating techniques, as well as screen printing and inkjet printing, have been used to deposit MXenes onto various substrates.^195,196^ A MXene subject to layer-by-layer assembly into heterostructures, with other 2D materials, is also an exciting prospect.^197^

## 6 Outlook

Despite the significant progress that has been made in the field of MXenes, there are still several challenges that need to be addressed before they can be widely used in practical applications. In 2020, experts in the MAX/MXene research area listed key issues for MXene research and development, as well as future opportunities and challenges in the field.^198^ This list is still relevant, containing 32 points, presented in the order of priority. Some of the main points will, if addressed properly, lead to new discoveries in the form of emerging structures and an expansion of nonoxide 2D materials. The latter will provide building blocks for future technologies. While significant research efforts are directed toward the realization of specific properties, ranging from superconductors and topological insulators to ferromagnets, the materials performance is also given extensive attention. Improvement thereof is achieved by, for example, optimization of the synthesis procedures, functionalization for property enhancement, as well as identification of new pathways for property tuning (such as control of morphology, assembly into heterostructures, etc.) to meet specific application requirements.

As the field of MXenes continues to grow, the development of suitable methods for accelerated discovery of new MXenes, with unique properties, remains a crucial future challenge. Addressing this challenge will be critical for unlocking the full potential of MXenes and facilitating their adoption in fields beyond those investigated today and will require collaboration between researchers from different research disciplines. In the sections below, we therefore discuss challenges related to the topic of the present Review, atomic scale design of MXenes and their parent materials, from both theoretical and experimental perspectives.

### 6.1 Future Prediction of Novel MAX Phase Precursors

If one considers the traditional top-down approach for MXene synthesis, i.e., selective etching of a 3D precursor, there are numerous opportunities for future MXene development. The recent addition of (Mo_0.8_Nb_0.2_)_5_AlC_4_^70^ and high-entropy MAX phases^71−73,75−77,99−107^ has opened up avenues for higher-order MAX phases, where theoretical studies could be used for identifying promising candidates for MXene parent materials and reveal whether chemical order or solid solutions can be expected. Here, choice of method for structure predictions is crucial, also including machine learning procedures for more efficient predictions.^199−204^ Such procedures should ideally also consider formation enthalpy as a descriptor for more realistic predictions. Another aspect is simultaneous solid solution on multiple sites, e.g., on M and A, such as in (Zr_1–*x*_Nb_*x*_)_2_(Al_1–*y*_Sn_*y*_)C^205^ and (Zr_1–*x*_Ti_*x*_)_2_(Al_0.5_Sn_0.5_)C,^206^ which can be used as a path to access novel elemental combinations. Here theory could be used to identify the specific elements needed to achieve stable materials over which range of compositions.

For double-metal MAX phases that display chemical order, it has been experimentally demonstrated that M′ and M′′ do not always necessarily have a strict ratio, with tendencies for intermixing.^113^ This means a deviation from an ideal M′/M′′ ratio.^97,108,113^ This, in turn, could provide novel compositions or impact the stability of the material. For example, for the (Mo_2/3_Sc_1/3_)_2_AlC *i*-MAX/Mo_3/4_C MXene, it has been shown that the MAX phase can accommodate a higher Sc content than the ideal 2:1 ratio for Mo/Sc. This is, however, not translated into the corresponding MXene, which simply dissolves upon attempted etching. Predicting new MXene precursors or modifying existing ones evidently requires analysis of whether or not 3D to 2D conversion is possible, in line with section 6.2. Another pathway is to move beyond MAX phases into related materials such as *M*_*n*_Al_3_C_*n*+2_ and *M*_*n*_Al_4_C_*n*+3_, or double-layer Ga (as in Mo_2_Ga_2_C) and explore possibilities for double-metal solid solution disorder and order.

With emerging new routes for MXene synthesis, future predictions of MXene precursors require additional methods reaching beyond the evaluation of formation enthalpy, which has been successful to date. This is evident from the recently reported structural modification of MAX phases by various intercalants, which in turn realizes MXenes composed of elements beyond those used traditionally.^207^ Notably, many of the more recently discovered MAX phases, where the A layer is based on, e.g., Au, Cu, Fe, Co, and Ni, are obtained by elemental replacement of, for example, Al or Si through molten salt or noble metal substitution. The original phase before replacement is typically thermodynamically stable if evaluating the formation enthalpy, whereas the modified phase is not.^24^

In the quest for identifying new MXene precursors, an outstanding challenge has been to expand the chemical space of X in MXene beyond C and N to B, to get what has been referred to as MBenes. While MAX phases based on B have been reported,^208^ no 3D to 2D conversion has been achieved for these materials. Instead, a 2D transition metal boride, Mo_3/4_B_2–*x*_T_*z*_ (boridene/MBene),^209^ was synthesized by selective etching of a so-called *i*-MAB phase,^210^ which can be seen as a B-based equivalent of *i*-MAX phases, though of a slightly different composition. MAB phases have a larger palette of structural variations than MAX phases, from M_*n*+1_AlB_2*n*_,^211^ with a single layer of Al, and MAlB with double layers of Al,^211^ to M_4_AlB_4_ with double layers of M.^212^ The prospects of chemical conversion of these materials and similar compounds into their 2D counterparts is intriguing, but at the same time it challenges the definition of what constitutes a MXene or a MXene-related material. The future will show how the definition evolves based on the development of the field of MXene research.

### 6.2 Future Prediction of MXene Derivation

The understanding of MXene synthesis is becoming increasingly profound, with the development of more realistic theoretical models. We are currently able to mimic conditions that are relevant for both electrochemical and conventional etching in HF. But there are still several outstanding answers to be provided by theoretical work in the future. For example, one is to answer how easily different multilayered MXenes can be delaminated into single-layer 2D MXenes. Another important aspect is describing alternative etching protocols, in particular those using molten salt as the etchant, as there have been several recent high-profile studies illustrating how the MXene family could be extended by the use of molten salt etchants. Understanding this would involve the development of chemical potentials that are valid for the chemical environment in the presence of the molten salt. Furthermore, understanding the transformation from multilayers to single layers may be most relevant here, as many examples have demonstrated the formation of multilayered MXenes using molten salt.

In the case of molten salt etching, one may ask whether the same criteria are valid as for HF, or if the mechanism is so fundamentally different that the problem has to be approached differently. If the former were to be true, it would be enough to express the chemical potentials in the environment of a molten salt. However, even if one sets apart the different chemical reactions taking place, the molten salt etching has another fundamental difference from conventional HF etching: The etching takes place at elevated temperatures. This is a necessity in order to have a molten salt, but one may hypothesize whether the temperature also affects the etching, as a higher temperature gives access to a larger part of the potential energy surface and, as a consequence, an increased mapping of possible structures. As discussed in section 3.3.3, in the case of HF etching, the free energy of vacancy formation of A and M elements is a decent descriptor to predict if a MAX phase can be selectively etched or not. For example, the overall thermodynamically favorable etching of Mo_2_GaC is hindered due to the initial part of the etching being endergonic.^168^ Such a scenario may not necessarily present an obstacle at elevated temperatures, but this would also mean that it is crucial that the formed MXene is thermodynamically stable against disintegration by the molten salt, which is not the case in HF.

The multilayer transformation of MAX phases into MXenes will require thorough theoretical investigations. Considering the several recent examples of MXenes formed in multilayers using molten salt etching, the proof of delamination is conspicuous by its absence, and it is relevant to question whether all multilayer MXenes are possible to delaminate into single layers or not. One role of theory could be to compare if the choice of transition metal affects the interactions between MXene layers or if this is solely determined by the surface terminations. Within this lies the characterization of the bond type between the layers: if the interactions are mainly characterized by nonlocal London dispersion forces (commonly referred to as van der Waals forces) or whether electrostatic Coulomb forces also are important as a result of internal multipole moments within the MXene layers. The question is probably to what extent, rather than if, electrostatic interactions are important for the interlayer bonding considering they have a considerable role even for weakly interacting graphene sheets.^213^

Etching in acid or molten salt are not the only imaginable possibilities. Future theoretical studies will also have to consider how to treat etching in bases. The considerations do not necessarily need to be that different from the case of acid. The main difference is what species to include in the chemical potential depending on what base it used. However, this would also be the case if the theoretical considerations were to be extended to acids other than HF.

Notably, the theoretical considerations are not only important to understand MXene synthesis and to predict which parent MAX phases could be selectively etched using a particular synthesis protocol. It is anticipated that the knowledge base built for MXene synthesis will eventually be utilized to study the etching of other laminated 3D materials. The first material family to come to mind is probably transition metal borides, given their large variety in chemical composition at the same time as being structurally similar to MAX phases, combined with the recent demonstration of the 2D boridene (MBene).^209^ Future studies are, however, not limited to these materials, and we may envision computational screening studies of etching of layered materials in general, based on the acquired knowledge of MXene synthesis, showing why it is necessary that the understanding is as accurate as possible. Needless to say, other material families may possess challenges not encountered for MXenes, and to think that the same etching rules apply for all materials may be naive.

In section 3.4 we discussed the potential of using machine learning to elucidate patterns in data and develop criteria for synthesizability. However, it was evident that the features that the algorithm considered of highest importance for such predictions were not consistent with what has been learned from first-principles calculations. Machine learning will certainly play an important role for future materials discovery, but the quality and relevance of the data fed to the machine learning machinery is probably of essence. Another direction for machine learning is to make use of the vast amount of computed data existing in publications and databases. A challenge is how to compare data obtained with different levels of theory or numerical precision. An outstanding challenge is whether machine learning, or artificial intelligence, could be utilized to compare data obtained in different studies. This is, however, not specific for MXenes but rather a problem that computational materials science will have to face sooner rather than later.

### 6.3 Prospects for MXene Synthesis and Processing

One of the main challenges for future applicability of MXenes is the development of environmentally friendly, safe, and efficient synthesis methods. Maybe the most important aspect is methods that allow for production of large quantities, also for MXenes beyond those based on Ti, which are by far the most investigated MXenes to date. While this is an area where there are a lot of research activities,^192,214,215^ allowing synthesis of up to 10 kg MXene per batch, even larger quantities are required to widen the applicability of these materials even further. Also, specific synthesis methods produce material with a surface chemistry decided by the terminations inherent to the choice of synthesis technique, which in turn is decisive for the materials properties. Hence, both synthesis and functionalization in the form of post-processing will likely require specialized equipment and expertise for different MXenes in different forms.

While MXene properties and tuning thereof is not summarized in the present Review, it is known that the MXene characteristics originate from structure, composition, and morphology, as well as defects. Control of the latter is dependent on the choice of synthesis procedure. Tuning of defect concentration can be achieved through alloying, intentional introduction of ordered and disordered vacancies,^82,84,88^ and process parameters such as etching time, chemical concentration, and temperature.^216^ For improved control of properties, this is an area where more research activities are expected, targeting both an increase and a reduction of defects, ideally for various morphologies and for attainable sizes of MXene sheets.

Among the outspoken challenges related to MXene synthesis and processing is the development of self-assembly techniques for preparation of films with aligned flakes (sheets), and with control of both the flake orientation and distance between adjacent flakes. This is of particular importance for applications relying on intercalation/deintercalation, such as energy storage and environmental applications. New methods in this area are also of importance for the utilization of MXenes as nanoscale building blocks, which in turn will allow for the development of nanoarchitectures including horizontally/vertically aligned sheets and the formation of highly controlled composites and heterostructures. Use of MXenes together with other 2D materials in heterostructures presents outstanding prospects, as MXenes can be the building blocks that provide, e.g., the conductive, electrochemical, or catalytic properties. Predictive theoretical methods for property evaluation combined with controlled assembly of materials may open pathways for unique properties and devices. This also requires control of junctions between different materials and matching of their work functions, to control the interaction and bonding between the components and to be able to select the combination of properties required for a specific application.

We end this Review by stressing the advantages and opportunities arising from the integration of computational and experimental approaches to design novel MAX phases (and related laminated materials) and MXenes. Basic evaluation of the phase stability of MAX phase chemistries serves not only to identify new materials but also to guide experimentalists in the choice of synthesis procedures based on whether the 3D material is stable. For example, solid state synthesis in the form of powder sintering allows for verifying experiments that target thermodynamically stable compounds. Thin-film processing, on the other hand, allows for the formation of metastable structures far from thermodynamic equilibrium. Whether stable or metastable 3D phases, they may be converted to 2D through selective etching, with further modification possible through, e.g., treatment with molten salt. Also here, theory-guided experiments will allow faster progress of MXene development, and this is expected to go hand-in-hand with the development of theoretical pathways that more accurately approximate the experimental etching procedures, as outlined above. One important aspect that requires more attention is how to handle metastable phases or transition states, and this is an area where AI may contribute to moving the research area forward. Another crucial aspect of MXene synthesis is delamination. While this is not important for all applications, hampered delamination possibilities may reduce the overall applicability of the material. To date there are limited fundamental understanding and no intrinsic rules that can explain why specific intercalation/delamination solvents work for some MXenes and not others. While the size and polarity of small cations and larger molecules, respectively, have been suggested to be of importance for MXene intercalation, fundamental studies of this topic are more or less uncharted territory. This is an area where extensive and systematic theoretical exploration of weakly bonded MXene multilayers can contribute with valuable information that provides insight into reactions that take place on small length scales during short time intervals. Altogether, the outlook for MXenes and related materials is very promising and has the potential to transform various fields through the broad range of exceptional properties. If the research and development will continue to expand at the current pace, MXenes are likely to play a key role in the development of future advanced materials and technologies.
